# An altered extracellular matrix–integrin interface contributes to Huntington’s disease-associated CNS dysfunction in glial and vascular cells

**DOI:** 10.1093/hmg/ddac303

**Published:** 2022-12-22

**Authors:** Sarah J Hernandez, Ryan G Lim, Tarik Onur, Mark A Dane, Rebecca Smith, Keona Wang, Grace En-Hway Jean, Andrea Reyes-Ortiz, Kaylyn Devlin, Ricardo Miramontes, Jie Wu, Malcolm Casale, David Kilburn, Laura M Heiser, James E Korkola, David Van Vactor, Juan Botas, Katherine L Thompson-Peer, Leslie M Thompson

**Affiliations:** Department of Neurobiology and Behavior, University of California Irvine, Irvine, CA 92697, USA; Institute for Memory Impairments and Neurological Disorders, University of California Irvine, Irvine, CA 92697, USA; Department of Molecular and Human Genetics, Baylor College of Medicine, Houston, TX, USA; Jan and Dan Duncan Neurological Research Institute at Texas Children’s Hospital, Houston, TX, USA; Genetics & Genomics Graduate Program, Baylor College of Medicine, Houston, TX 77030, USA; Department of Biomedical Engineering, OHSU, Portland, OR 97239, USA; Department of Biomedical Engineering, OHSU, Portland, OR 97239, USA; Department of Neurobiology and Behavior, University of California Irvine, Irvine, CA 92697, USA; Department of Developmental and Cell Biology, University of California, Irvine, CA 92697, USA; Department of Biological Chemistry, University of California Irvine, Irvine, CA 92697, USA; Department of Biomedical Engineering, OHSU, Portland, OR 97239, USA; Institute for Memory Impairments and Neurological Disorders, University of California Irvine, Irvine, CA 92697, USA; Department of Biological Chemistry, University of California Irvine, Irvine, CA 92697, USA; Department of Neurobiology and Behavior, University of California Irvine, Irvine, CA 92697, USA; Department of Biomedical Engineering, OHSU, Portland, OR 97239, USA; Department of Biomedical Engineering, OHSU, Portland, OR 97239, USA; OHSU Knight Cancer Institute, Portland, OR 97239, USA; Department of Biomedical Engineering, OHSU, Portland, OR 97239, USA; OHSU Knight Cancer Institute, Portland, OR 97239, USA; Department of Cell Biology, Harvard Medical School, Boston, MA 02115, USA; Department of Molecular and Human Genetics, Baylor College of Medicine, Houston, TX, USA; Jan and Dan Duncan Neurological Research Institute at Texas Children’s Hospital, Houston, TX, USA; Genetics & Genomics Graduate Program, Baylor College of Medicine, Houston, TX 77030, USA; Quantitative & Computational Biosciences, Baylor College of Medicine, Houston, TX 77030, USA; Department of Developmental and Cell Biology, University of California, Irvine, CA 92697, USA; Reeve-Irvine Research Center, University of California, Irvine, CA 92697, USA; Center for the Neurobiology of Learning and Memory, University of California, Irvine, CA 92697, USA; Sue and Bill Gross Stem Cell Research Center, University of California Irvine, Irvine, CA 92697, USA; Department of Neurobiology and Behavior, University of California Irvine, Irvine, CA 92697, USA; Institute for Memory Impairments and Neurological Disorders, University of California Irvine, Irvine, CA 92697, USA; Department of Biological Chemistry, University of California Irvine, Irvine, CA 92697, USA; Center for the Neurobiology of Learning and Memory, University of California, Irvine, CA 92697, USA; Sue and Bill Gross Stem Cell Research Center, University of California Irvine, Irvine, CA 92697, USA; Department of Psychiatry and Human Behavior, University of California Irvine, Irvine, CA 92697, USA

## Abstract

Astrocytes and brain endothelial cells are components of the neurovascular unit that comprises the blood–brain barrier (BBB) and their dysfunction contributes to pathogenesis in Huntington’s disease (HD). Defining the contribution of these cells to disease can inform cell-type-specific effects and uncover new disease-modifying therapeutic targets. These cells express integrin (ITG) adhesion receptors that anchor the cells to the extracellular matrix (ECM) to maintain the integrity of the BBB. We used HD patient-derived induced pluripotent stem cell (iPSC) modeling to study the ECM–ITG interface in astrocytes and brain microvascular endothelial cells and found ECM–ITG dysregulation in human iPSC-derived cells that may contribute to the dysfunction of the BBB in HD. This disruption has functional consequences since reducing ITG expression in glia in an HD *Drosophila* model suppressed disease-associated CNS dysfunction. Since ITGs can be targeted therapeutically and manipulating ITG signaling prevents neurodegeneration in other diseases, defining the role of ITGs in HD may provide a novel strategy of intervention to slow CNS pathophysiology to treat HD.

## Introduction

Huntington’s disease (HD) is a hereditary, monogenic disease caused by a CAG expansion in the N-terminus of the huntingtin (*HTT*) gene (1). An expansion of 40 or more CAG repeats produces pathogenic forms of the HTT protein (mutant HTT, mHTT), which induces a cascade of molecular pathologies that leads to behavioral changes, cognitive decline and choreic movements (2). Although the direct genetic cause of the disease is known, disease-modifying therapies for HD do not yet exist.

While HD is primarily thought of as a disease that affects neurons, non-neuronal cell types, such as glia and brain endothelial cells, also contribute to pathophysiology (3–9). The most abundant glial cell type of the CNS is astrocytes (10). The contribution of astrocytes to HD pathology is supported by recent studies showing that astrocytes play a role in altering glutamate signaling and function in an HD patient-derived stem cell model (11,12) and HD patient postmortem tissue (13). HD astrocytes also contribute to neuronal maturation deficits by reducing electrophysiological response (11), increasing autophagic responses in neurons (14) and increasing neuroinflammation (15). Consistent with these findings, our recent studies (12) using single-nuclei RNA sequencing (snRNAseq) analysis of induced pluripotent stem cell (iPSC)-derived astrocytes (iAstros) found transcriptomic changes in HD astrocytes suggestive of abnormal activation of signaling pathways associated with glutamate signaling, synaptic transmission, actin cytoskeleton and cellular adhesion.

Like astrocytes, HD-associated alterations have been described in brain microvascular endothelial cells (BMECs) that make up the blood–brain barrier (BBB) (16,17) and postmortem HD tissue shows brain capillary leakage (16), aberrant angiogenesis (16,18) and BMEC degeneration through disrupted tight junction (TJ) protein expression (16). These phenotypes are also observed in the transgenic R6/2 HD mouse model (16,18). We, and others, have used iPSC models of BMECs (iBMECs) to demonstrate intrinsic angiogenic impairments, reduced drug efflux capacity and increased paracellular and transcellular permeability (17,19).

The extracellular matrix (ECM) of the brain is produced, in part, by astrocytes (20). The ECM serves to support astrocytes and BMECs that make up the vasculature in the brain and contribute to the integrity and selective permeability of the BBB. The integrity of the BBB is essential for protecting brain neurons from toxic molecules in the blood stream and a leaky BBB can contribute to neurodegeneration. There is evidence that the ECM is altered in HD based on studies in the striatum and cortex of YAC128 (transgenic full-length HTT) and R6/2 mice (transgenic fragment model), in which the activity of matrix metalloproteinases (MMPs), which act to restructure the ECM, are increased (21). In HdhQ111 striatal cells, reduced MMP expression decreased toxic HTT fragment accumulation and prevented cell death and MMP loss-of-function *Drosophila* alleles suppressed HD-associated climbing deficits and photoreceptor degeneration (21). To date, the ECM composition in HD has not been directly studied in human tissue.

Both astrocytes and BMECs express integrins (ITG), which anchor these cells to the ECM and play an important role in maintaining the BBB. ITGs are a large family of heterodimeric transmembrane receptors that mediate cell–matrix adhesion and communication through bidirectional signaling (22,23). Expression of ITGs is perturbed during pathological states of the CNS, and ECM defects are well documented in patients of stroke, multiple sclerosis and Alzheimer’s disease (AD) and matrix changes localize with degenerative pathology, such as β-amyloid plaques and angiopathy in AD (24). In a rat stroke model, post-injury administration of the ECM molecule osteopontin (SPP1) protected against BBB disruption, improved neurobehavior and stimulated autophagy in neurons (25). There is major interest in the pharmaceutical industry in developing therapeutic approaches targeting ITGs to treat a range of medical conditions, with 117 ITG-targeting molecules in active or completed clinical trials (clinicaltrials.gov) and 6 receiving FDA approval (26,27). Thus, if ITGs play a critical role in neurodegeneration in HD, safe and well-tolerated ITG therapeutics could be repurposed to treat HD.

While leveraging the matrix–ITG interface appears to be broadly beneficial for neurodegenerative disease, our understanding of the role of ITG and dysregulation of the ECM in HD is limited. A genome-wide, *in vivo* genetic screen showed that the ITG signaling pathway is a modifier of neuronal *mHTT* toxicity in R6/2 mice (28) and proteomic changes in the ITG signaling pathway were found in the superior frontal gyrus from HD patients (29). Additionally, snRNAseq from human HD cingulate cortex demonstrates some astrocyte populations are represented by gene expression changes related to cell–matrix adhesion (30). These studies suggest that the ECM and possibly ITG signaling is altered in HD. However, the relationship between the ECM and ITGs in the context of HD pathogenesis has not been defined and the therapeutic potential for modulating ITG expression has not been explored.

Here, we used transcriptomic data sets to define a profile of ECM-associated and ECM-interacting genes in HD iBMECs and iAstros that may cause dysregulation of the ITG signaling pathway. Functional assessment by altering ECM substrates *in vitro* demonstrated that HD iBMECs exhibit aberrant interactions with the ECM that weakens barrier fidelity and contributes to degenerative pathology. Using a *Drosophila* model to mechanistically investigate the contribution of ITG function in HD, we showed that reducing ITG expression, specifically in glial cells, suppresses HD-associated CNS deficits. Taken together, our work identifies deficits at the ECM–ITG interface that contribute to molecular and cellular HD pathogenesis in non-neuronal cell populations that can be leveraged to suppress CNS dysfunction. Such findings add to the growing body of evidence that non-neuronal cells participate in HD pathogenesis and identify the ITG signaling pathway in these cell types as a potential target for therapeutic intervention.

## Results

### The HD matrisome is altered at the transcriptomic level in non-neuronal cell types

The complete complement of extracellular proteins in the ECM is referred to as the matrisome (31). To begin to investigate if the matrisome is dysregulated in HD, we assessed transcriptomic data sets previously described from our group for both astrocytes and brain microvascular cells differentiated from patient and control iPSCs (12,17).

#### Astrocytes

We first analyzed snRNA-seq on iAstros differentiated from unaffected individuals (with 18Q and 33Q) and adult-onset (<60Q) HD-affected individuals (with 46Q and 53Q) to define transcriptional signatures that may guide disease-altered astrocytic function in HD (12). This analysis identified maturation deficits, impairment of drivers of glutamate signaling and alterations in adhesion- and cytoskeleton-related genes as top biological processes changed in HD iAstros. Relating to the HD matrisome, increased expression of ECM markers, adhesion-related ITGs, collagens and fibronectin (FN1) was also seen in HD-enriched cell clusters (12). These data highlight novel alterations associated with the activation of ECM- and adhesion-related genes in HD iAstros. We therefore further investigated these data to fully characterize HD-associated matrisome dysregulation in astrocytes.

**Figure 1:**
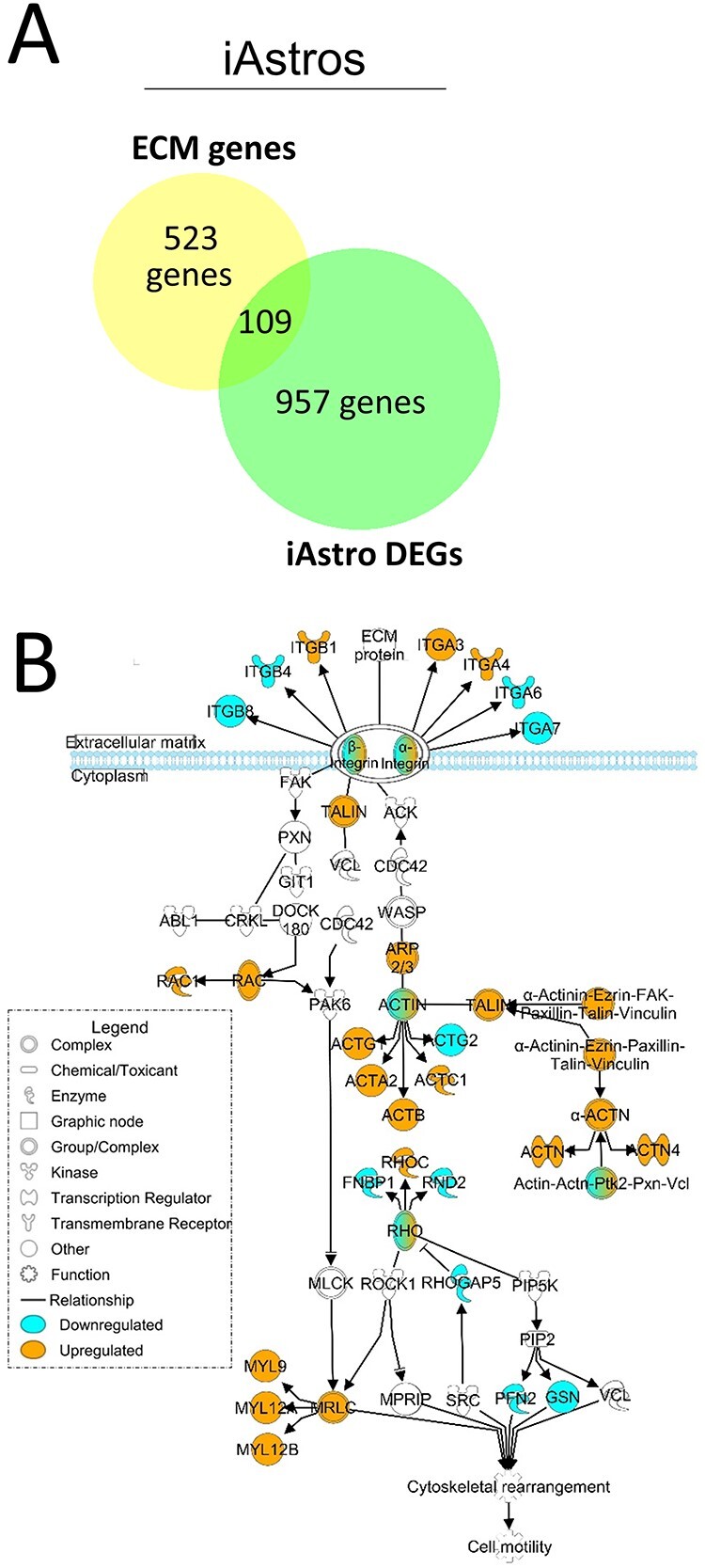
Matrix-associated and matrix-interacting genes are dysregulated in HD iAstros. (**A**) Overlap of iAstro DEGs with ECM genes expected to be expressed in astrocytes (6.87% of DEGs were ECM-related compared with 3.34% of ECM-related genes found within iAstros; chi = 112.8, *P* < 0.0001; *N* = 1, *n* = 2). Chi square with Yate’s correction. (**B**) IPA of the ITG signaling pathway overlaid with the RNAseq DEG list comparing HD with control iAstros. Blue genes are downregulated; orange are upregulated. *N* = 2.

Comparing the 1065 differentially expressed genes (DEGs) from HD iAstros with a list of 632 matrix-related genes expected to be expressed in astrocytes, we identified 109 overlapping matrix-related genes (Fig. 1A). While matrix-related genes make up 3.34% of genes expressed by iAstros, matrix-related genes make up 6.87% of iAstro DEGs, demonstrating a significant enrichment of ECM genes within the iAstro DEGs (chi = 112.8, *P* < 0.0001).

The original analysis of the iAstro snRNAseq data identified several ITGs (*α6*, *α11*, *β1*, *β4* and *β8*) as dysregulated in HD-enriched cell clusters (12). Therefore, we further examined the full ITG signaling pathway using Ingenuity Pathway Analysis (IPA) to gain insight into potential signal transduction dysfunction that may result from an HD-associated aberrant matrisome–ITG interface in astrocytes. In HD iAstros, the ITG signaling pathway is highly dysregulated (Fig. 1B). Along with dysregulation of ITGs at the plasma membrane, talin and members of the actin family are upregulated. Talin physically links transmembrane-spanning ITGs to the actin cytoskeleton to mediate the forces that bind the cell to the extracellular space (32). Cytoskeletal-regulated locomotion is controlled, in part, by the assembly and disassembly of ITG-containing focal adhesions at the leading edge of the cell (33). As such, alterations in ITG expression and/or function participate in the regulation of cytoskeletal activation for cellular locomotion. When assessed in the context of the ITG signaling pathway, our analysis indicates that this cytoskeletal activation may occur downstream of disease-associated alterations at the matrisome–ITG interface.

**Figure 2:**
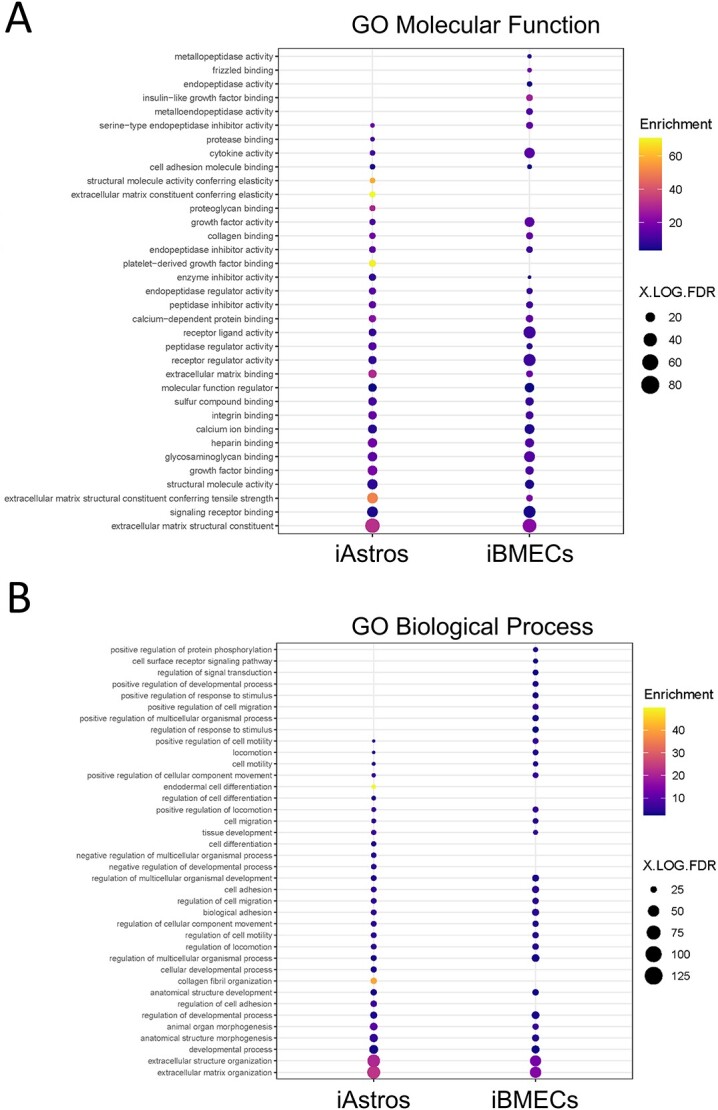
Aberrant matrisome contributes to HD pathogenesis. GO analysis of biological functions (**A**) and processes (**B**) showing enrichment for terms with known association to HD pathogenesis in iAstros (left) and iBMECs (right). Figure made using GOrilla comparing a background list of genes expected to be expressed in specified cell types with genes of the matrisome that were dysregulated in HD compared with control samples. Gene enrichment was plotted.

We then performed GO enrichment analysis (GOrilla) (34,35) for Molecular Functions (Fig. 2A) and Biological Processes (Fig. 2B) on matrix-related genes represented in the iAstro DEG list. Supplementary Material, Table S1 contains gene lists and GO terms associated with the analysis, which provided additional details regarding which specific ECM and adhesion biological processes and functions the dysregulated matrisome genes may be involved. We identified terms related to increased collagen expression, increased expression of adhesion-related genes and activation of the actin cytoskeleton (Supplementary Material, Table S1).

#### Brain microvascular endothelial cells

**Figure 3:**
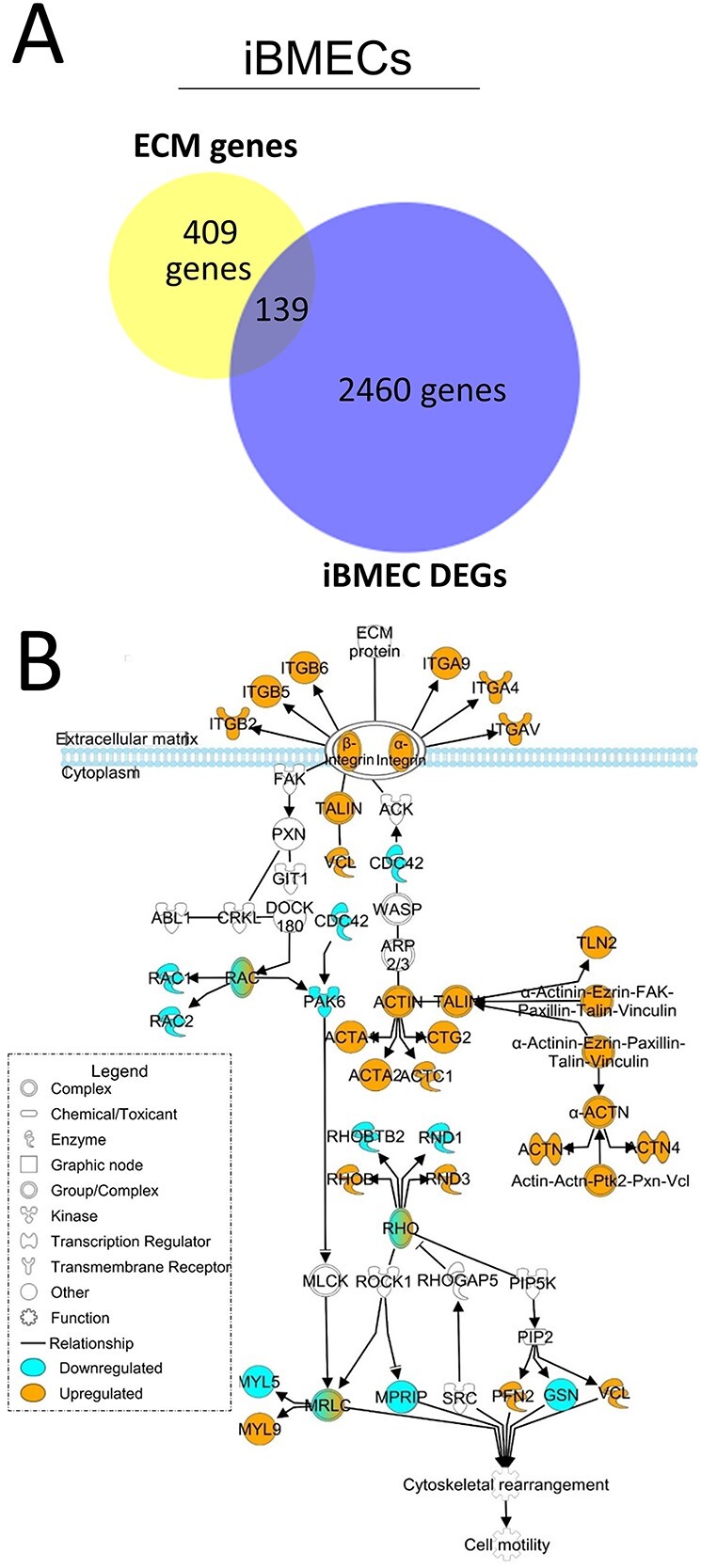
Matrix-associated and matrix-interacting genes are dysregulated in HD iBMECs. (**A**) Overlap of iBMEC DEGs with ECM genes expected to be expressed in BMECs (5.37% of DEGs were ECM-related compared with 4.41% of ECM-related genes found within iBMECs; chi = 3.85, *P* < 0.0497; *N* = 2, *n* = 2). Chi square with Yate’s correction. (**B**) IPA of the ITG signaling pathway overlaid with the RNAseq DEG list comparing HD with control iBMECs. Blue genes are downregulated; orange are upregulated. *N* = 2.

Astrocytes are critical for laying down ECM protein for both neurons and vascular cells (20), including collagen, which we found to be one of the top GO terms in our HD iAstros. To further define HD-associated matrisome dysregulation and connect HD astrocyte matrix-related DEGs to the matrisome of HD vascular cells, we next examined transcriptomic data from HD and control iBMECs (17). BMECs are surrounded by an ECM-rich basal lamina contributed by astrocytes and pericyte that supports and dictates barrier integrity (36,37). Examining transcriptional dysregulation of the matrisome in these cell types may help to define molecules contributing to HD-associated barrier function, which may be connected to the matrix-related DEGs we identified in our HD iAstros. RNAseq data (17) of iBMECs differentiated from two iPSC lines from unaffected individuals (with 28Q and 33Q) and four iPSC lines from juvenile (>60Q) HD-affected individuals (with 60Q, 66Q, 71Q and 109Q) were analyzed. Comparing the 2599 DEGs with a list of 441 matrix-related genes expected to be expressed in this cell type, we identified a significant dysregulation of 139 matrisome-related genes (Fig. 3A). While 4.41% of genes expressed by iBMECs are made up of matrix-related genes, 5.37% of the DEGs are comprised of matrix-related genes, demonstrating that iBMEC DEGs also have an enrichment of ECM genes resulting from HD (chi = 3.85, *P* < 0.0497).

When the 139 dysregulated matrisome genes were analyzed using IPA, the ITG signaling pathway emerged as being highly dysregulated (Fig. 3B). Dysregulation of the ITG pathway in both iAstros and iBMECs suggests that similar mechanisms may be driving ITG impairment in these cell types. Examination of this pathway reveals the upregulation (orange) of signaling components at the plasma membrane, including various ITG and key components of focal adhesions (talin, vinculin, actin and actinin) that form the macromolecular structures to mechanically link cells to the ECM. Dysregulation of focal adhesion components may explain adhesion and motility differences previously described in HD iBMECs (17). Furthermore, dysregulation of ITG signaling genes in both juvenile- and adult-onset lines suggests that it is a common pathological feature caused by expanded mHTT.

Matrix-related genes represented in the iBMEC DEGs were used for GO enrichment analysis (34,35) (Supplementary Material, Table S1) of Molecular Functions (Fig. 2A) and Biological Processes (Fig. 2B). This analysis identified biological processes with known deficits in HD iBMECs, such as Angiogenesis, Positive regulation of cell migration, Positive regulation of locomotion and the Canonical WNT signaling pathway, consistent with angiogenic deficits identified in the study from which this data set was derived (17). GO analysis suggests matrisome dysregulation may contribute to these defects.

iBMEC GO analysis also identified terms associated with processes and functions that are well described as being dysregulated in HD (Fig. 2), suggesting a novel role for HD-associated matrisome dysregulation in these processes. Such terms include Positive regulation of transmembrane receptor protein serine/threonine kinase signaling pathway and Positive regulation of protein phosphorylation perhaps related to altered phosphorylation, that is well described in HD (38–42), Additionally, this analysis identified terms associated with autophagic balance (Regulation of cellular protein metabolic process and Regulation of proteolysis), a process deeply engrained in HD pathophysiology (43). The contribution of the matrisome to autophagy is an emerging topic, particularly in neurodegenerative disease (44). Full exploration of the synergistic cooperation of the ECM and autophagy remains to be investigated for HD.

Lastly, iBMEC GO analysis identified terms that suggest a deficit in the ability of the cell to signal with its extracellular environment (Cell adhesion, Regulation of cell migration and Collagen fibril organization). Identification of the term Collagen fibril organization perhaps further suggests a dysfunction in ITG-mediated signaling, as collagens are a primary ligand for this signaling pathway (45), particularly for BMECs, which require collagen produced by themselves and astrocytes for proper formation and function (46). This provides a novel analytical component for this data set, indicating an HD-associate deficit at the cell/matrix interface at the transcript level.

#### ITG dysregulation across cell types

For both iAstros and iBMECs, there was dysregulation of specific ITGs known to be expressed in those cell types (44). In iAstros, snRNAseq revealed increased expression of ITGs *α3* and *β1* and decreased expression of ITGs *α6*, *β4* and *β8*. In astrocytes, these ITGs use collagens (*α6* and *β1*), laminin (*α3*, *α6*, *β1* and *β4*), thrombospondin (THBS1) (*α3* and *β1*), FN1 (*β1* and *β8*), vascular endothelial growth factor (*β1*), SPP1 (*β1*), latency activated peptide (LAP)-TGF-β1 (*β8*) and vitronectin (VTN) (*β8*) as ligands (44). Additionally, ITGs *α4* and *α7* were identified as DEGs, but have not yet been described in this cell type (44). In iBMECs, bulk RNAseq revealed increased expression of ITGs *αV* and *α4*. In BMECs, these ITGs use FN1 (*αV* and *α4*), SPP1 (*αV* and *α4*), LAP-TGF-β1 (*αV*), THBS1 (*αV* and *α4*), fibrinogen (*αV*) and VTN (*αV*) as ligands. Additionally, ITGs *α9*, *β2*, *β5* and *β6* were identified as DEGs, but have not yet been described in this cell type (44). The only ITG that appeared as a DEG in both data sets was *α4*, which was upregulated in both iAstros and iBMECs. *ITGα4* has only been previously described as being expressed in BMECs; however, we also observe expression in our astrocytes. Interestingly, most of the terms that overlap between the iAstro and iBMEC GO analysis for biological process are centered around cell adhesion.

Together, these data suggest that there is an HD-associated transcriptional dysregulation both upstream of the plasma membrane with ECM-associated genes and downstream of the plasma membrane with a major pathway that uses the ECM for signaling—the ITG signaling pathway—in iAstros and iBMECs. The matrisome–ITG interface plays critical functional roles for directing biological process known to be disrupted in HD, such as cell motility that is important for astrocyte phagocytosis (47,48) and barrier function necessary for BBB fidelity in BMECs (17,49,50). The above data begin to suggest a synergistic role for matrisome dysregulation in both astrocytes and BMECs, such as collagen and FN1 dysregulation in HD astrocytes that could be causing altered ITG signaling and adhesion/cell motility deficits in HD BMECs. As such, defining matrix molecules, particularly those used as ITG ligands and secreted by astrocytes, on which barrier deficits are observed in HD iBMECs may identify targets for modulation that can be used to correct or compensate for pathogenic features leading to barrier deficits.

### ECM modulation establishes aberrant ITG interactions

The matrisome–ITG interface is critical for barrier function at the BBB. Barrier deficits, including reduced barrier function and morphological aberrations of TJs, were previously described using HD iBMECs (17). Considering the known role for the matrisome–ITG interface in barrier function, we sought to determine if ECM composition influences HD-related cellular deficits in HD iBMECs.

Two iPSC lines that we previously utilized for modeling HD BBB deficits, unexpanded (control, 33Q; CS83iCTR3n1) or expanded *HTT* (HD, 109Q; CS09iHD109n1), were differentiated into iBMECs (Supplementary Material, Fig. S1A) as described (46). These lines were chosen based on prior characterization, robustness and reproducibility of HD versus control phenotypes. As before, both lines were morphologically similar by phase contrast imaging throughout differentiation (Supplementary Material, Fig. S1B) and expressed known brain endothelial cell markers such as the glucose transport BMEC marker GLUT1 and TJ proteins, claudin 5 (CLDN5) and zona occludens 1 (ZO1) (Supplementary Material, Fig. S1C). Additionally, both lines formed functional barriers, as measured using transendothelial electrical resistance (TEER) (Supplementary Material, Fig. S2A). In line with previous descriptions of HD-associated barrier deficits in iBMECs, the HD line demonstrated feathered TJs (Supplementary Material, Fig. S1C), indicative of poor barrier fidelity and reduced TEER measurements compared with the control line (Supplementary Material, Fig. S1B), indicative of reduced barrier function.

**Figure 4:**
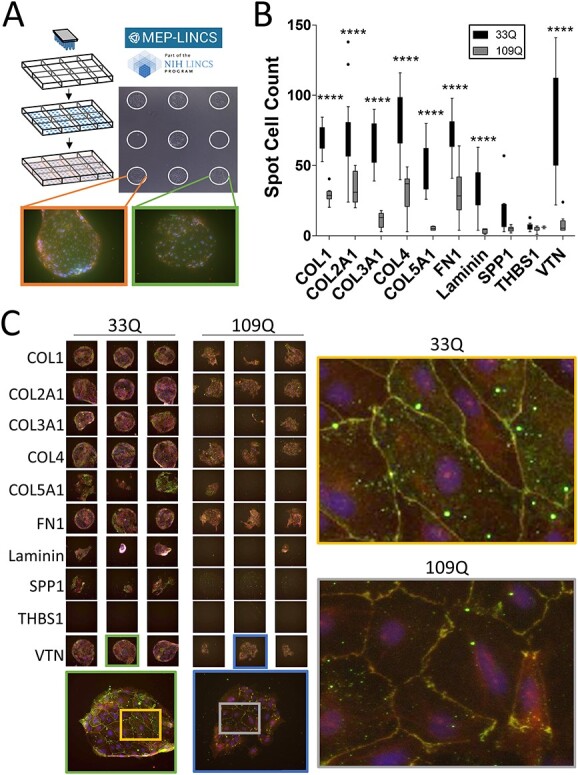
HD iBMECs have reduced adhesion and morphological deficits on ITG ligands. (**A**) MEMA schematic. Each spot is ~300 μm and is a unique ECM substrate plated at random on the array in multiple technical replicates. iBMECs are seeded, fixed and stained. Certain spots prevent TJ formation (green box), whereas others enabled TJ formation (orange box). (**B**) 33Q and 109Q iBMECs were assessed for adhesion by counting the number of Hoechst-positive cells per spot. All ITG ligands from the MEMA are shown. Two-way ANOVA with Bonferroni post-hoc. *N* = 1, n ≥ 11. (**C**) iBMECs stained for TJ proteins CLDN5 (green) and ZO1 (red) with a DAPI counterstain. Three representative technical replicates are shown. All technical replicates can be found in Supplementary Material, Figure S2. All ITG ligands shown (left). Morphological disruption of TJs is shown on VTN (right). *N* = 1, *n* ≥ 11. Each spot is ~300 μm.

To determine if ECM composition influences HD-related cellular deficits in HD iBMECs, the iBMECs were plated onto microenvironmental microarrays (MEMAs), a novel platform that assesses the effect of ECM modulation on biological endpoints in a high-throughput manner (51,52) (Fig. 4A). MEMAs are comprised of cell culture plates on which ECM molecules are printed in 300 μm spots, either individually or in combination, in an array format. Cells are then seeded onto MEMAs, allowing for the determination of how ECM proteins influence targeted cell behaviors and phenotypes. Since the microenvironment on each MEMA is controlled save for one variable, the ECM molecule, these arrays are an unambiguous tool for identifying ECM molecules that drive phenotypic changes. Thirty-eight unique ECM substrates (Supplementary Material, Fig. S3A and B), with an additional 10 in combination with collagen 1 or collagen 4 (COL4), were assessed (Supplementary Material, Fig. S4A and B). These substrates included ECM proteins whose genes were found to be dysregulated in HD iAstros at the RNA level. HD iBMECs had reduced adhesion on eight substrates, highlighted in Figure 4B, all of which are ITG ligands and many of which were dysregulated by RNAseq in both iBMECs and iAstros (Figs 1 and 3).

Of the ITG ligands assessed on the MEMA, four (FN1, SPP1, TBHS1 and VTN) were substrates used by ITGs already known to be expressed in BMECs and identified as DEGs in iBMECs and iAstros by RNAseq. While SPP1 and THBS1 overall had little cell adhesion of either control or HD iBMECs, control cells adhered well to both FN1 and VTN with HD iBMECs demonstrating reduced adhesion on these substrates. To assess the potential functional impact of ECM changes, iBMECs on MEMAs were stained for the TJ proteins CLDN5 and ZO1 to assess TJ morphology. On the ITG ligands with differential adhesion, including FN1 and VTN, HD iBMECs exhibited feathering of TJs, indicative of poor barrier formation and function, which was not the result of reduced cell number. Figure 4C highlights TJ disruption on ITG ligands with particular attention called to VTN. HD iBMEC adhesion deficits on VTN were subsequently validated using a CyQUANT adhesion assay (Supplementary Material, Fig. S2B). VTN-binding ITGs maintain transcytotic barrier function in endothelial cells (49) and modulate TJ protein expression and permeability in murine BMECs (50). Mouse embryos null for VTN-binding ITGs displays CNS vascular pathologies (53,54) and conditional CNS deletion causes progressive neurological phenotypes (seizure, rigid gait, axonal degeneration and early death) (55) similar to HD mouse models (56), potentially suggesting participation by aberrant VTN–ITG interaction in HD-associated CNS dysfunction. We were able to extract and quantify TJ staining data from the MEMA analysis for 7 of the 10 ITG ligands tested. Interestingly, on these matrices, HD iBMECs demonstrated increased expression of CLDN5 and ZO1 (Supplementary Material, Fig. S5). So, while HD iBMECs have decreased barrier function and feathered TJs, they also have increased TJ protein expression, perhaps as a compensatory mechanism to improve barrier function controlled by the pathological state.

These data indicate a functional dysregulation upstream of the ITG signaling pathway at the matrisome–ITG interface and suggest that ITG–ligand interactions contribute to barrier deficits in HD iBMECs. Given the therapeutic targetability of ITGs (26,27), understanding how ITGs and the matrisome functionally contribute to such HD-associated deficits may perhaps represent a targetable, molecular linchpin for HD-associated pathogenesis in non-neuronal cell types.

### Reduced ITG expression suppresses CNS deficits in HD flies

We next sought to define the contribution of ITGs to CNS function in HD at the organismal level using *Drosophila. Drosophila* represents an inexpensive, fast, genetically tractable model system that has previously been used to define compensatory and/or pathogenic mechanisms in HD (57,58). Additionally, 60% of fly genes have human orthologs (59) and ITGs are conserved from invertebrates to mammals. With this in mind, we tested the contribution that ITG signaling in non-neuronal cell types has on HD-associated CNS dysfunction *in vivo*.

We used a rapidly progressing *Drosophila* model of HD harboring a 231 amino acid N-terminal fragment with 128 glutamines (*HTT^231NT128Q^*) (60) using the GAL4-*UAS* system to drive expression of human *mHTT* in glia (*repo > GAL4*). *Repo* drives expression in all glia except those of the midline (61), which includes cell types analogous to astrocytes and BMECs. The *Drosophila* cell type most analogous to BMECs is subperineural glia (SPG), which form the outer-most cell layer surrounding the fly brain. Together with perineural glia, SPG make up the brain barrier in flies. Our MEMA data identified FN1 and VTN as matrices of interest in iBMECs that may have biological relevance for HD. However, flies have no obvious FN1 ortholog (62), so we first performed an assessment of the fly ortholog of VTN-binding ITGs, *inflated* (*if*), driven by *repo*. *if* is orthologous to ITGs *αIIb*, *α8*, *αV* and *α5*, all of which interact with β subunits to bind VTN except *α5* (62). These mammalian ITGs also use FN1 and SPP1 as ligands, with *αV* being expressed in BMECs, astrocytes and microglia, and *α5* being expressed in BMECs and astrocytes (44). We performed a widely used behavioral climbing assay that acts as a proxy for CNS function. Metrics such as climbing speed or distance can be assessed over time and scored. This is a robust assay where healthy flies will reliably climb to the top of a vial until motor performance is affected by age, whereas flies that express *mHTT* in either glia or neurons show deficits in their climbing ability when they are still relatively young (57). Using a custom robotic assay system that measures climbing speed as a function of time (57), we demonstrate that reduced *if* expression leads to a suppression of the CNS-associated behavioral deficit caused by *mHTT* expression in glia (Supplementary Material, Fig. S6). Based on these findings, we conclude that reducing function of VTN-binding ITGs may be a protective response in HD.

**Figure 5:**
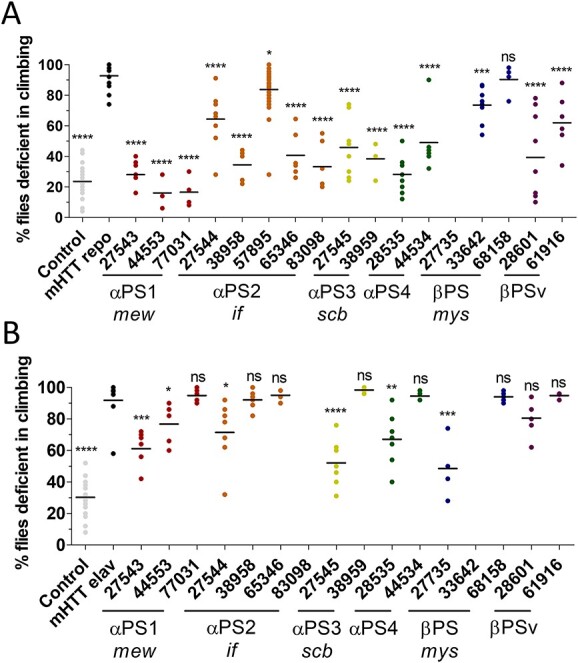
HD-associated CNS deficits are suppressed when ITG expression is reduced in glial, but not neuronal, cell populations. An HD fragment *Drosophila* model (HTT231NT128Q) was crossed with lines that reduce expression of various ITGs (*X*-axis) expressed using the Gal4 system. ITG orthologs are listed on the *X*-axis. Numbers represent BDSC stock numbers. A climbing assay was performed (**A**) at day 10 when mHTT was expressed in glia and (**B**) day 15 when mHTT was expressed in neurons. When mHTT was expressed in either glia (gray dots, A) or neurons (gray dots, B), most flies were unable to climb past 5 cm. When ITG mutants were crossed with flies that express mHTT only in glia (black dots, A), the climbing deficit was largely suppressed. When ITG mutants were crossed with flies that express mHTT only in neurons (black dots, B), the climbing deficit is less suppressed overall. Horizontal lines represent mean. One tailed, unpaired *t*-tests were performed comparing each line to the mHTT-expressing line within each data set. *P*-values for significant differences are: ^*^^*^^*^^*^P<0.0001, ^*^^*^^*^P = 0.001. (A) 57895 *P* = 0.0174, 68158 *P* = 0.3103. (B) 27543 *P* = 0.0002, 44553 *P* = 0.0409, 27544 *P* = 0.0195, 28535 *P* = 0.0044, 27735 *P* = 0.0006. All flies were raised at 25°C. *N* = 3–9 with 8–10 animals per replicate, *n*=5.

Our iBMEC MEMA data also suggest that there are HD-associated deficits with other members of the ITG family. Therefore, we next asked how reduced expression of other members of the ITG family may influence HD-associated CNS function and if these effects differ in neurons compared with glia. We crossed HD flies with RNAi lines that reduce the expression of each ITG (Fig. 5). While the fly ITGs *αPS3* (*scab*, *scb*), *αPS4* and *βPSv* have no clear vertebrate orthologs, other fly ITGs are orthologous to mammalian ITGs (62). *Multiple edematous wings* (*mew, αPS1*) is orthologous to ITGs *α3*, *α6* and *α7* (62), which use laminin and THBS1 as ligands and are expressed in BMECs and astrocytes (*α3* and *α6*), as well as neurons (*α3*, *α6* and *α7*) (44). *Myospheroid* (*mys, βPS1*) is orthologous to *ITGβ1* (62), the primary mammalian β ITG. *ITGβ1* uses almost all ITG ligands as substrates, including collagens, laminins, SPP1, THBS1 and VTN, with expression in various cell types of the brain, such as BMECs, astrocytes, neurons and pericytes (44). We crossed flies expressing *mHTT* either in glia (*repo > GAL4*) or neurons (*elav > GAL4*) with RNAi flies to knock down expression of various ITGs. Using the climbing assay, we developed a screen to quantify the percentage of flies unable to climb to or beyond 5 cm within 10 s. Timepoints were selected that produced a severe climbing deficit in HD flies (Supplementary Material, Fig. S7); flies expressing *mHTT* in glia climbed on day 10 post-eclosion and those expressing *mHTT* in neurons climbed on day 15 post-eclosion, but still showed overall survival to enable detection of both exacerbation and suppression of the climbing phenotypes. Overall, we found that reduced expression of various ITGs predominantly suppressed the CNS-associated behavioral deficit when *mHTT* is expressed in all glia (Fig. 5A). However, the same is not generally true when *mHTT* is expressed in neurons; reducing the expression of various ITGs did not significantly suppress the climbing defect phenotype (Fig. 5B).

Data for a subset of these lines were additionally validated with *mHTT* expressed in either glia or neurons at both days 10 and 15 (Supplementary Material, Fig. S8). Confirming that one needs to evaluate the glial crosses at the earlier day 10 timepoint (Supplementary Material, Fig. S8A) to observe any exacerbation of phenotypes, flies expressing *mHTT* in glia were all dead by day 15 (Supplementary Material, Fig. S8B). Flies expressing *mHTT* in neurons had an attenuated climbing deficit at day 10 (Supplementary Material, Fig. S8C) compared with day 15 (Supplementary Material, Fig. S8D). Additionally, when HD flies are crossed to ITG mutants, suppression of the climbing deficit is observed at both timepoints regardless of *mHTT* expression in glia or neurons. Taken together, these data demonstrate that reduced function of ITGs in non-neuronal cells consistently modulates CNS functions, primarily in a protective manner, leading to improved behavior (climbing).

## Discussion

Previous studies have suggested a link between alterations in the ECM and HD pathogenesis, including evidence of aberrant cell migration/motility (17,63), impaired brain barrier fidelity (16–18,64) and progressive neurological decline (65) in both animal models and HD postmortem tissue. We present a novel finding that the ITG family has a role in pathogenesis and CNS dysfunction in HD. Since ITGs are therapeutically targetable (26,27) and manipulation of ITG-mediated signaling through ECM modulation prevents neurodegeneration and stimulates autophagy in other disease models (66,67), defining the role of ITGs in HD may provide a targeted mechanism of intervention to correct or compensate for neurodegeneration in HD.

To study the role of ITGs in HD, we first reanalyzed published transcriptomic data sets to explore the hypothesis that ECM–ITG interactions contribute to HD pathogenesis. We analyzed snRNAseq from iAstros and bulk RNAseq from iBMECs to identify dysregulation at the ECM–ITG interface that may contribute to cell autonomous deficits for each cell type. In both cell types, ITGs known to be expressed in each cell type were identified as DEGs (44). We identified expression changes in ITGs and their endogenous ligands in the HD cells. Of note, we saw expression changes in collagen and FN1 in HD astrocytes, which are ligands also used by BMECs for proper development and BBB formation.

The ECM is critical for BBB fidelity as well as for anchoring BMECs to maintain barrier integrity. ITGs expressed on BMECs attach these cells to the ECM. Using iBMECs as a model, we tested how modulating the ECM, using MEMAs, influences well-characterized HD barrier deficits. HD iBMECs grown on the MEMAs demonstrated reduced adhesion on eight matrices, all of which were ITG ligands. Four ITG ligands assessed on the MEMA are known to be expressed in BMECs and were identified in iBMECs by RNAseq as DEGs—FN1, SPP1, TBHS1 and VTN. Of these, control iBMECs adhered well to FN1 and VTN, whereas HD iBMECs had reduced adhesion to these substrates. Additionally, HD iBMECs on FN1 and VTN had feathered TJs, indictive of poor barrier formation and function. Concomitantly, we observed increased TJ protein expression on these matrices. This perhaps suggests a compensatory mechanism where the cell increases the expression of TJ proteins to improve barrier deficits known to coincide with feathered junctions (17). It is also possible that feathered junctions increase the area of junctions between cells, which would also increase levels of the TJ proteins that span these junctions. These results are consistent with our iBMEC RNAseq data, which identified the ITG *ITGαV* expressed in BMECs as a DEG and *ITGαV* that uses VTN as a ligand.

These findings are of interest because it is known that VTN is a critical factor for barrier fidelity (49,50,53–55) and recently the VTN–ITG interface was identified as a key regulatory mechanism for inhibiting transcytosis in BMECs (68). Modulation of VTN either through genetic ablation or mutation, as well as BMEC-specific ITGα5 deletion, is sufficient for causing BBB leakage through increased transcytosis in mice (68). Increased transcytosis has been reported in striatal tissue from the R6/2 HD mouse model (16) as well as HD iBMECs (17). Our work showing reduced HD iBMEC adhesion to VTN provide insight for a contributing mechanism that may underlie barrier function pathology in HD. Considering HD astrocytes shows a reduction in FN1 expression, this finding suggests that the dysfunction of ITGs may directly contribute to BBB deficits in HD.

To better understand the potential cell-type-specific causal or compensatory role and to further determine the functional impact of ITG modulation on pathophysiology, we genetically reduced the expression of selected ITGs in an HD *Drosophila* model and determined the impact on climbing behavior as a readout for CNS function. Of note is that the *Drosophila* model harbored an N-terminal fragment of mHTT, whereas patient-derived iPSC models contained full-length, endogenous mHTT. As such, these flies represent a rapidly progressing model for HD. When ITG expression was specifically reduced in glial cell populations, the HD-associated climbing deficit was suppressed. The ameliorative effect of selectively reducing ITGs in neurons on this HD-associated behavior was less pronounced. One interpretation for these results relates to cell population ratios—if ITG expression is modulated in a far larger cell population it may stand to reason a larger effect would be observed. However, unlike humans where about 90% of the nervous system is comprised of glia, only about 15% of the fly nervous system is made up of glia (69). Alternatively, these cell type differences may be because of variations in the types, levels and functions of ITGs in neurons compared with glia: neurons express 4 ITG dimers *(α3β1*, *α6β1*, *α6β4* and *α7β1*) that bind to three ITG ligands, whereas glial cell types express 19 ITG dimers [eight in BMECs (*α1β1*, *α3β1*, *α4β1*, *α5β1*, *α6β1*, *α6β4*, *αVβ1* and *αVβ3*), seven in astrocytes (*α1β1*, *α3β1*, *α5β1*, *α6β1*, *α6β4*, *αVβ5* and *αVβ8*) and four in microglia *(αLβ2*, *αVβ3*, *αVβ5* and *αVβ8*)] and make use of 10 out of 12 ITG ligands (44). This may suggest a greater role and influence of ITG signaling in glia than neurons in HD and that regulating ITG expression specifically in glia may be beneficial in reducing HD-associated pathology. Together, these findings indicate that the interaction of ITGs on astrocytes and BMECs with the ECM is impaired in HD and that resetting ITG–ECM interactions may be beneficial in restoring BBB integrity in the disease.

While transcript expression of various ITGs is increased, our MEMA data suggest that ITG function is decreased. Since we show that reduced ITG function suppresses HD-associated CNS dysfunction in flies, these findings could be interpreted in three ways: (i) ‘Reduced ITG transcript expression does not equate to changes at the protein level’. This could be accounted for by differences in protein turnover, posttranslational modifications or other aspects of protein metabolism. (ii) ‘The changes we observe are compensatory’. Our MEMA data demonstrate reduced adhesion, which suggests reduced ITG function. Concomitantly, we demonstrate that reducing ITG expression in HD flies, particularly in glia, suppresses HD-associated CNS dysfunction. One could reason that reduced ITG function is a biological attempt to compensate for HD pathology and further reducing ITG levels, and thus function, provides additional improvement to the system. Indeed, flies have previously been used to define compensatory and pathogenic genes in HD (57,58). If reducing expression of a pathogenically downregulated gene exacerbates *mHTT*-induced toxicity, it could suggest that the lower expression of that gene in HD is pathogenic. Conversely, if reducing expression of a pathogenically downregulated gene decreases *mHTT*-induced toxicity, one coule reason that the altered expression of that gene in HD is compensatory. The latter seems to be the case for our data. (iii) ‘Cell-type-specific changes may be occurring that are pathogenic in one cell type and compensatory in another’. In the case of iBMECs, it could be that increased ITG transcript expression does not translate to increased function and reduced ITG activity is potentiating barrier deficits associated with these cells. It is also possible that the reduced ITG function is still compensatory in the BMECs but not sufficient to compensate for the barrier deficits, whereas ITG RNA levels are increasing because of a feedback mechanism. At the same time, reduced ITG function in glial cell types could act as a protective mechanism, and when ITG function is further reduced, CNS dysfunction associated with the disease is suppressed. These points highlight the need for a better understanding of how compensatory and pathogenic effects may contribute cell-type-specific effects in HD. Additionally, the progressive nature of HD underscores the importance of understanding temporal changes of these effects throughout disease progression. Such an understanding would aid in the development of treatments that could be adjusted throughout disease course.

The ability of ITG knockdown to suppress HD behavioral phenotypes in the HD *Drosophila* model is reproducible and was replicated in three separate labs across the country, at Baylor College of Medicine in Houston, TX, at University of California, Irvine in Irvine, CA, and at Harvard Medical School in Boston, MA. This rigorously supports our conclusion that reducing ITG expression in glial cell populations suppresses HD-associated CNS dysfunction in an HD *Drosophila* model and clearly identifies the ITG signaling pathway as having potential therapeutic relevance for HD. ITG targeted drugs have been developed to treat a number of proliferative disorders (26,27). They are generally well tolerated and six ITG targeted drugs have received FDA approval (26,27). In preclinical mouse studies, ITG ligands were also found beneficial in stroke models and rescued BBB disruption (25). Thus, we propose that targeting this family of molecules may have therapeutic benefit for HD and should be explored in detail further.

## Materials and Methods

### Pathway analysis

RNA sequencing of iBMECs was performed as previously descried (17). Transcriptomic data were analyzed using QIAGEN’s Ingenuity® Pathway Analysis (IPA®, QIAGEN Redwood City, www.qiagen.com/ingenuity). GOrilla was used to for GO network analysis (http://cbl-gorilla.cs.technion.ac.il/) (34,35).

### Maintenance of iPSCs and iBMEC differentiation

iPSC lines were generated as described (70). All lines were cultured on hESC-qualified Matrigel (Corning) using mTeSR culture medium (STEMCELL Technologies) and passaged using Versene (Gibco) at 70–80% confluency. iBMECs were differentiated as described (46). Briefly, when iPSC reached 70–80% confluency, they were washed with PBS−/− (Gibco). Accutase (Fisher Scientific) was added and cells were incubated at 37°C for 8–10 min until cells were completely singularized. Cells were dislodged from the plate using trituration with mTeSR supplemented with 10 μm ROCK inhibitor (Y-27632; STEMCELL Technologies). Following centrifugation, cells were counted and plated at a density of 3.5e4 cells/cm^2^. Daily media changes were performed for 2 days. On the third day, cells were fed with DeSR1 [DMEM/F12 (Invitrogen 11330-032) supplemented with 0.5% GlutaMAX (Invitrogen), 1% NEAA (Invitrogen) and 0.1 mm β-mercaptoethanol (Gibco)] supplemented with 6 μm CHIR99021 (Tocris Bioscience)—this begins day 0 of the differentiation protocol. For the next 5 days, daily media changes were performed with DeSR2 [DeSR1 medium supplemented with 2% B27 (ThermoFisher 17504044)]. On day 6, cells were fed with hESCR1 [hESFM (Invitrogen 11111), 20 ng/mL bFGF2 (R&D Systems), 2% B27] supplemented with fresh 10 μm retinoic acid (Sigma R2625) (RA). A full media change was performed on day 7 with hESCR1 with fresh RA. On day 7, culture dishes were coated with collagen IV (Sigma C5533) and FN1 (Fisher CB-40008) and incubated overnight at 37°C. The following morning, the collagen/FN1 was rinsed from culture plates with PBS−/− prior to media addition. On day 8, cells were subcultured with TrypLE (Fisher Scientific) and incubated for at 37°C 15 min. Cells were dislodged from the plate using trituration in TrypLE. hESCR1 was used to collect cells prior to centrifugation. Cells were plated onto collagen/FN1-coated culture dishes at a density of 1e6 cells/cm^2^ [except on transwells (see TEER methods)] in hESCR1 with fresh RA. Daily media changes were performed for 4 days with hESCR2 (hESFM with 2% B27). Phase contrast images were taken daily using an EVOS Cell Imaging system (Invitrogen).

### Immunofluorescence

On day 11 of the iBMEC differentiation protocol, cells for analysis by immunocytochemistry were washed with PBS+/+ and fixed according to antibody specifications. For TJ proteins CLDN5 and ZO1, cells were incubated with 95% ice-cold ethanol on ice for 30 min followed by 80% ice-cold acetone at room temperature for 1 min. For the transport protein GLUT1, cells were incubated in 100% ice-cold methanol on ice for 15 min. All cells were washed with PBS+/+ and blocked with 1% bovine serum albumin, 10% goat serum and 0.05% Triton-X. Primary antibodies were incubated overnight at 4°C (CLDN5 1:250, LifeTech 35-2500; ZO1 1:250, LifeTech 617300; GLUT1 1:200, Thermo A5-11315). Cells were washed three times with PBS+/+ prior to incubation with appropriate AlexaFlour secondaries (Life Technologies) at 1:1000 for 1 h at room temperature in the dark. Cells were counterstained with Hoechst for nuclear visualization. Quality control analysis images were taken using a Nikon Ti-E.

### Transendothelial electrical resistance

On day 8 of the iBMEC differentiation, 2e6 cells were plated on 0.4 μm transwells (Corning) in hESCR1 with fresh RA. As a quality control metric to ensure barrier formation, TEER measurements were taken every 24 h following subculture on day 8 for 5 days using an EVOM2 voltohmmeter with STX2 probes (World Precision Instruments). TEER measurements were normalized to resistance (Ω × cm^2^) across a collagen/FN1-coated transwell with an equal volume of media, containing no cells.

### Plating on MEMAs

On day 8 of the iBMEC differentiation protocol, MEMA plates were blocked with 1% F108 (BASF) for 20–30 min at room temperature. Blocking solution was gently aspirated and plates were rinsed twice with PBS+/+. 3e5 cells were plated onto blocked and rinsed MEMAs with hESCR1 with fresh RA and incubated at 37°C for 2 h. After incubation, plates were rinsed three times with hESCR1 to remove excess cells. hESCR1 with fresh RA was added and MEMA plates were incubated at 37°C for 3 days. MEMA plates were rinsed with PBS+/+ and fixed for immunofluorescence staining as described above. Analysis was performed at Oregon Health & Science University as described (52,71).

### CyQUANT adhesion assay

Flat-bottomed 96-well plates (Fisher Scientific) were coated with substrates according to manufacturer instructions. Matrices used were COL4 (Fisher Scientific CC077), FN1 (Fisher Scientific 1918-FN-02M) and VTN (Fisher Scientific A14700). Plates were stored at 4°C until used. Prior to use, plates were washed once with PBS−/− and blocked for 25–30 min at room temperature with 2% bovine serum albumin (Fisher Scientific 15260-037). Plates were washed three times with PBS−/−. Cells were grown to day 8 of the iBMEC protocol above and 143 K cells were plated per well. Cells were allowed to adhere for 30 min at 37°C. Following incubation, cells were flicked out of plate and wells were washed three times with PBS+/+. Excess PBS+/+ was removed and cells were stored at −80°C until processing. Plates were allowed to thaw at room temperature prior to processing with CyQUANT (Thermo C7026) according to manufacturer instructions. Briefly, 100 μL CyQUANT GR dye/cell lysis buffer was added per well. The solution was gently triturated over cells and allowed to incubate for 2–5 min at room temperature protected from light. Sample fluorescence was read at an excitation of 480 nm and emission of 520 nm.

### Fly husbandry

Fly lines were either generated as described (57) or obtain from the Bloomington *Drosophila* Stock Center (BDSC) or Vienna *Drosophila* Stock Center (VDSC) as shown in Table 1. *mHTT* was expressed using a pan-neuronal (*elav*) or a pan-glial (*repo*) driver. Flies were maintained at 23 or 18°C on standard media (molasses, yeast extract and agar).

**Table 1:** List of fly lines used as described in Materials and Methods. Fly lines were either generated as described previously or obtained from the BDSC or VDSC

**Gene**	**Genotype**	**Source**	**ID number**
*HTT* Frag elav	Xelav Yhid; +; HTT [231NT-128Q]/TM6B gal80	Botas lab	
*HTT* Frag repo	w; +; HTT [231NT-128Q]—repo-gal4/gal 80-TM6B	Botas lab	
	Nontargeting hpRNA	VDSC	13974
ITGαPS1 (*mew*)	y[1] v[1]; P(y[+t7.7] v[+t1.8] = TRiP.JF02694)attP2	BDSC	27543
	y[1] sc[^*^] v[1] sev[21]; P(y[+t7.7] v[+t1.8] = TRiP.HMS02849)attP2	BDSC	44553
	y[1] sc[^*^] v[1] sev[21]; P(y[+t7.7] v[+t1.8] = TKO.GS00808)attP40	BDSC	77031
ITGαPS2 (*if*)	y[1] v[1]; P(y[+t7.7] v[+t1.8] = TRiP.JF02695)attP2	BDSC	27544
	y[1] sc[^*^] v[1] sev[21]; P(y[+t7.7] v[+t1.8] = TRiP.HMS01872)attP40/CyO	BDSC	38958
	y[1] w[^*^] Mi(y[+mDint2] = MIC)if[MI12214]	BDSC	57895
	y[1] sc[^*^] v[1] sev[21]; P(y[+t7.7] v[+t1.8] = TRiP.HMC06096)attP40	BDSC	65346
	y[1] v[1]; M(v[+t1.8] = WKO.P1-D4)ZH-86Fb	BDSC	83098
ITGαPS3 (*scb*)	y[1] v[1]; P(y[+t7.7] v[+t1.8] = TRiP.JF02696)attP2	BDSC	27545
	y[1] sc[^*^] v[1] sev[21]; P(y[+t7.7] v[+t1.8] = TRiP.HMS01873)attP40	BDSC	38959
ITGαPS4	y[1] v[1]; P(y[+t7.7] v[+t1.8] = TRiP.HM05021)attP2	BDSC	28535
	y[1] sc[^*^] v[1] sev[21]; P(y[+t7.7] v[+t1.8] = TRiP.HMC02928)attP40	BDSC	44534
ITGβPS (*mys*)	y[1] v[1]; P(y[+t7.7] v[+t1.8] = TRiP.JF02819)attP2	BDSC	27735
	y[1] v[1]; P(y[+t7.7] v[+t1.8] = TRiP.HMS00043)attP2	BDSC	33642
	y[1] w[^*^] P(ry[+t7.2] = neoFRT)19A; P(w[+mC] = UAS-mys.L)3/TM3	BDSC	68158
ITGβPSv	y[1] v[1]; P(y[+t7.7] v[+t1.8] = TRiP.HM05089)attP2	BDSC	28601
	y[1] v[1]; P(y[+t7.7] v[+t1.8] = TRiP.HMJ23472)attP40	BDSC	61916

### Climbing assay

Female virgin flies carrying the *mHTT* transgene or the control line (VDSC 13974) were crossed to males carrying experimental alleles. Animals were raised and maintained at 25°C. The climbing assay was performed only on female progeny. In Houston, TX, the climbing assay assessed speed as a function of time and was carried out using a one-of-a-kind, custom robotic system as described (57). In Irvine, CA, and Boston, MA, the climbing assay quantified the percent of flies able to climb past 5 cm in 10 s at room temperature. Anesthetized with carbon dioxide, female progeny were sorted into experimental vials without food in groups of 8–10 flies with 4–20 replicates (Irvine, CA) or in groups of 5–10 flies with 2–6 replicates (Boston, MA) (biological replicates). Negative geotaxis was elicited by tapping flies to the bottom of the vials with three rapid succession taps, three times, over 2 s. The flies were allowed to climb for 10 s, and an image was taken against a white background with a mark parallel to the lab bench at 5 cm from the bottom of the vial. Following a 1-min recovery period, the trial was repeated. Each trial was repeated for a total of five times (technical replicates). In Irvine, CA, flies expressing *mHTT* in glia were climbed at day 10 and those expressing *mHTT* in neurons were climbed at day 15. In Boston, flies expressing *mHTT* in glia and neurons were climbed at both days 10 and 15.

**Abbreviations**
ACTN, actinin: AD, Alzheimer’s disease
  BBB, blood–brain barrier
  BMECs, brain microvascular endothelial cells
  CLDN5, claudin 5
  COL1, collagen 1
  COL4, collagen 4
  DEGs, differentially expressed genes
  BDSC, Drosophila Stock Center
  ECM, extracellular matrix
  FGF, fibroblast growth factor
  FN1, fibronectin
  GFAP, Glial fibrillary acidic protein
  HTT, huntingtin
  HD, Huntington’s disease
  iPSC, induced pluripotent stem cell
  *if*, *inflated*
  ITG, integrin
  iBMECs, iPSC models of BMECs
  iAstros, iPSC-derived astrocytes
  (LAP)-TGF-β1, latency-activated peptide
  MMPs, matrix metalloproteinases
  MEMAs, microenvironmental microarrays
  *mew*, *multiple edematous wings*
  mHTT, mutant HTT
  *mys*, *myospheroid*
  SPP1, osteopontin
  RA, retinoic acid
  *scb*, *scab*
  snRNAseq, single-nuclei RNA sequencing
  SPG, subperineural glia
  THBS1, thrombospondin
  TJ, tight junction
  TEER, transendothelial electrical resistance
  VEGF, vascular endothelial growth factor
  VDSC, Vienna Drosophila Stock Center
  VCL, vinculin
  VTN, vitronectin
  ZO1, zona occludens 1

## Supplementary Material

Supp_Fig_1_ddac303

Supp_Fig_2_ddac303

Supp_Fig_3_ddac303

Supp_Fig_4_ddac303

Supp_Fig_5_ddac303

Supp_Fig_6_ddac303

Supp_Fig_7_ddac303

Supp_Fig_8_ddac303

HMG-2022-CE-00614_Hernandez_Supp_Table_1_ddac303

HMG-2022-CE-00614_R1_Supplemental_Data_(web_posting_only)_ddac303
