# Supplementary figures and images for: An altered extracellular matrix–integrin interface contributes to Huntington’s disease-associated CNS dysfunction in glial and vascular cells
Source: Hum Mol Genet. 2022 Dec 22;32(9):1483–96. doi: 10.1093/hmg/ddac303 (PMC10117161; doi:10.1093/hmg/ddac303)

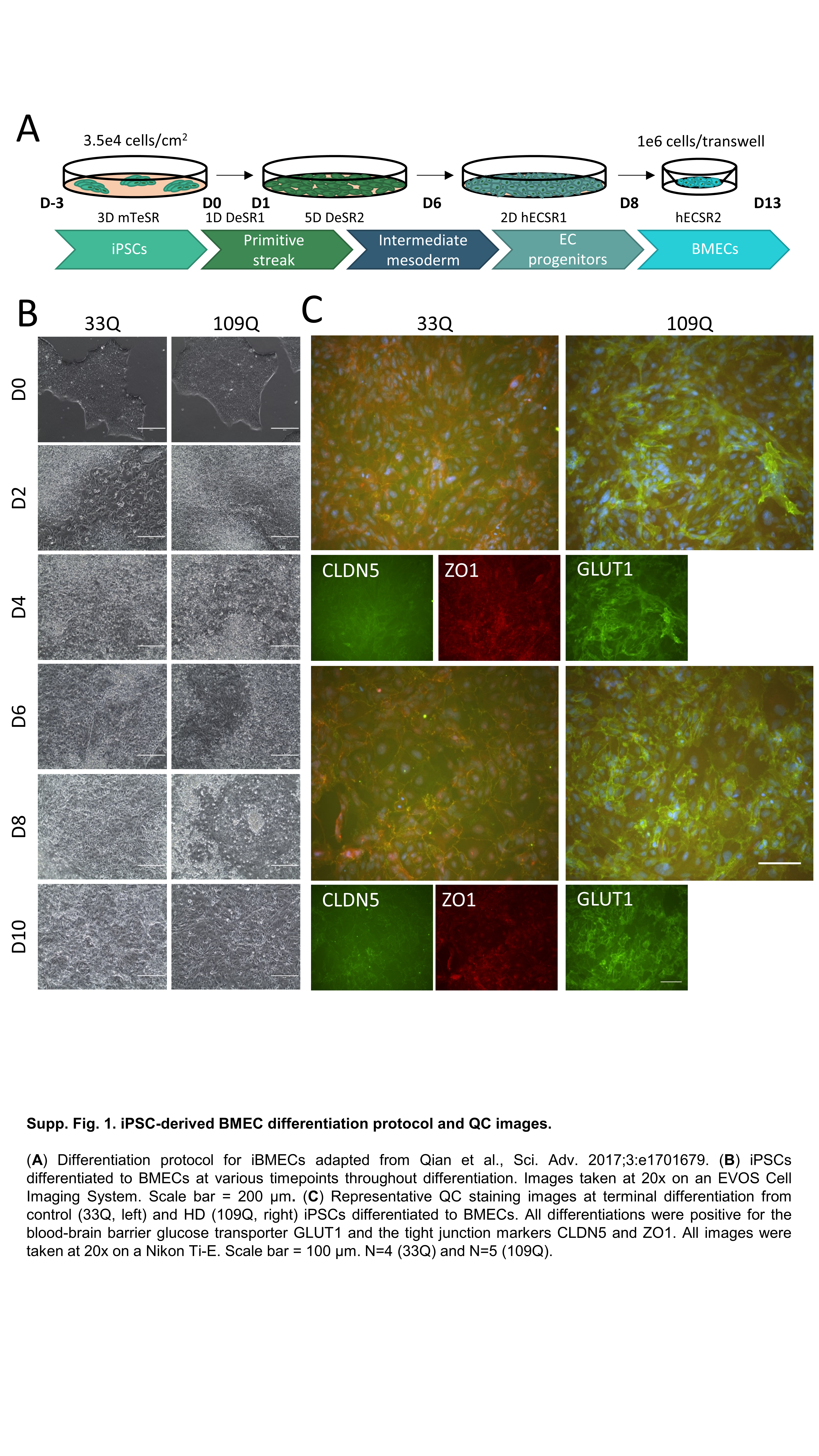

Supplement: Supp_Fig_1_ddac303 [file supp_fig_1_ddac303.zip › Supp_Fig_1_ddac303.TIF]

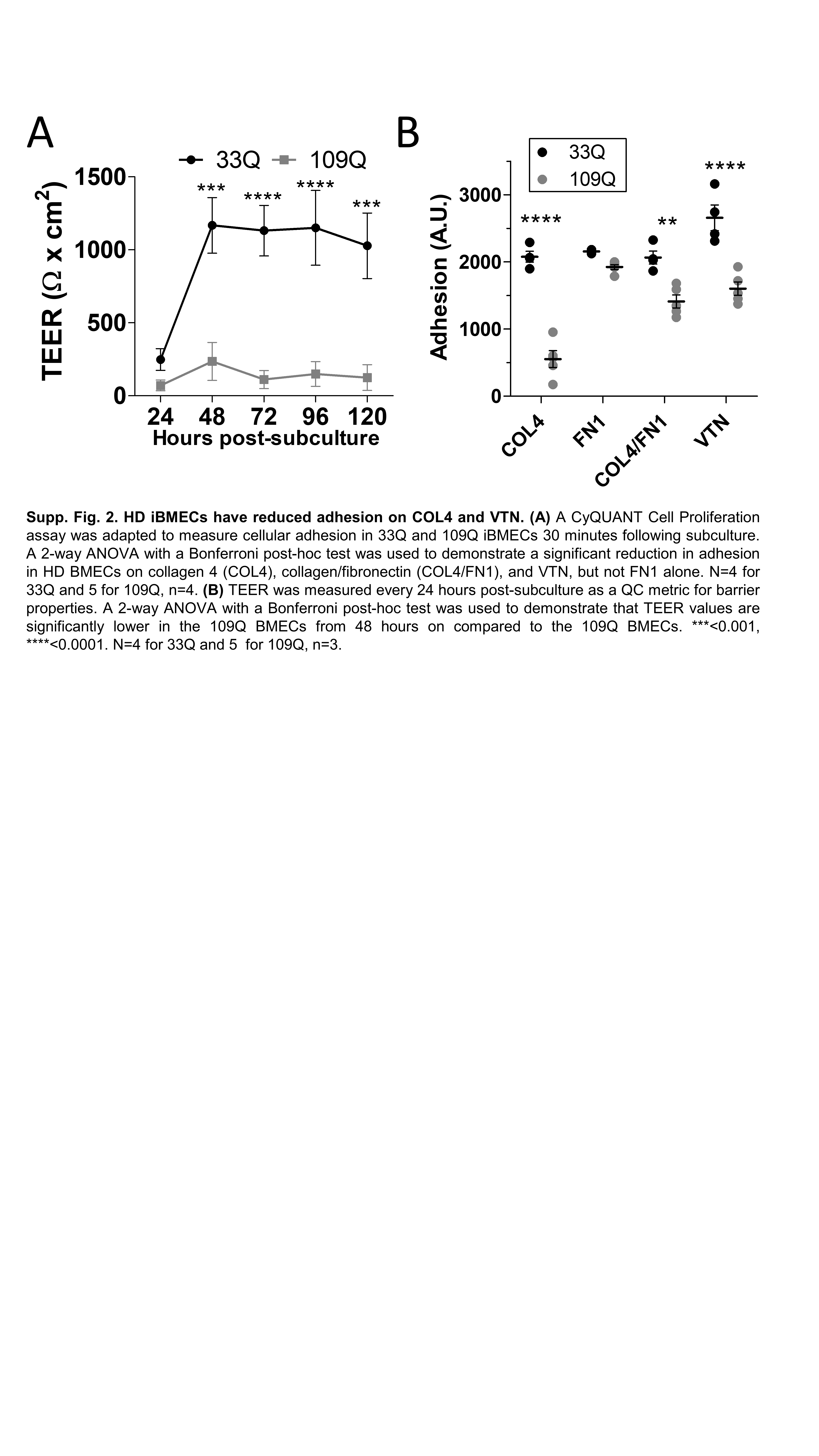

Supplement: Supp_Fig_2_ddac303 [file supp_fig_2_ddac303.zip › Supp_Fig_2_ddac303.TIF]

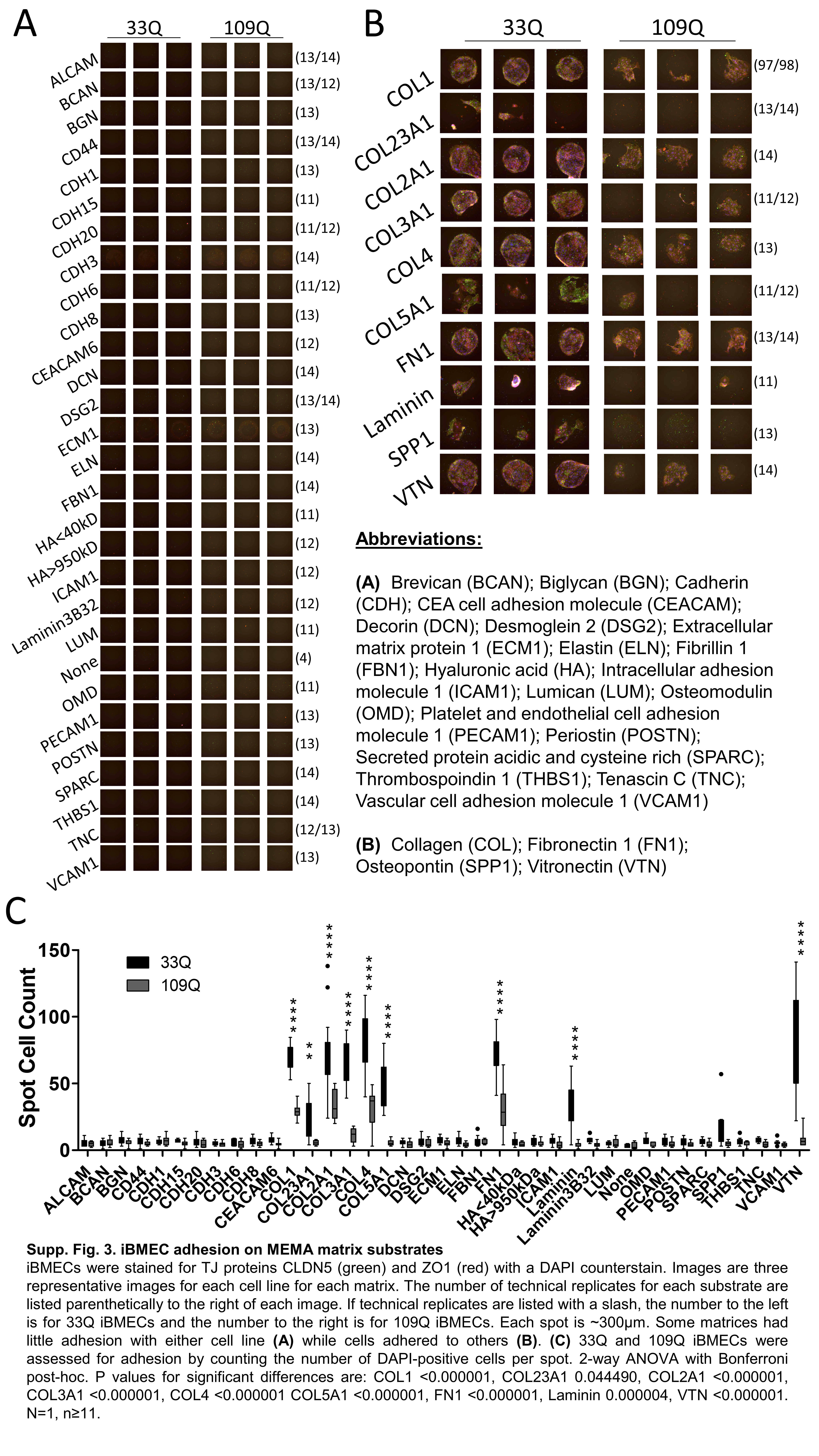

Supplement: Supp_Fig_3_ddac303 [file supp_fig_3_ddac303.zip › Supp_Fig_3_ddac303.TIF]

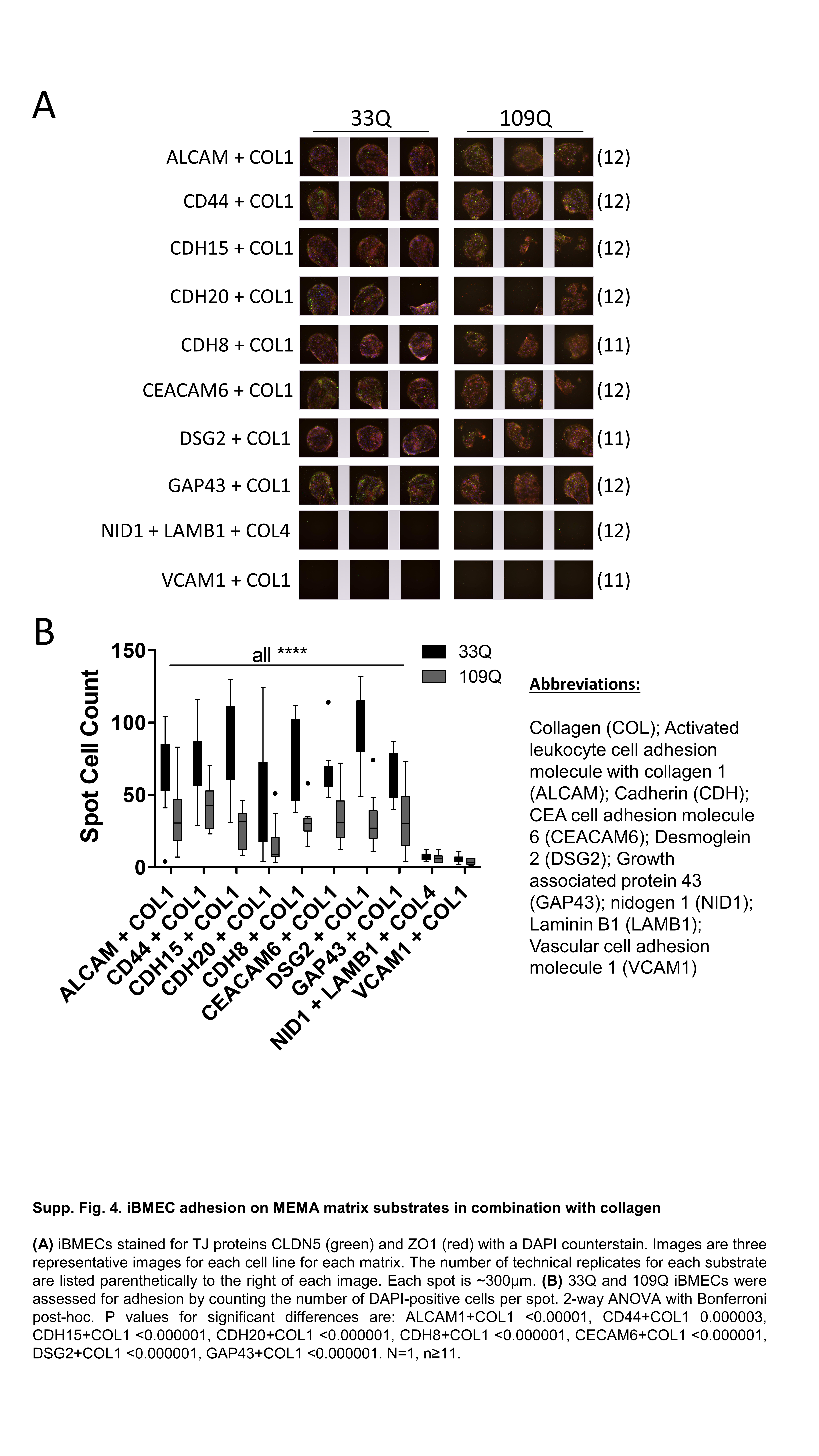

Supplement: Supp_Fig_4_ddac303 [file supp_fig_4_ddac303.zip › Supp_Fig_4_ddac303.TIF]

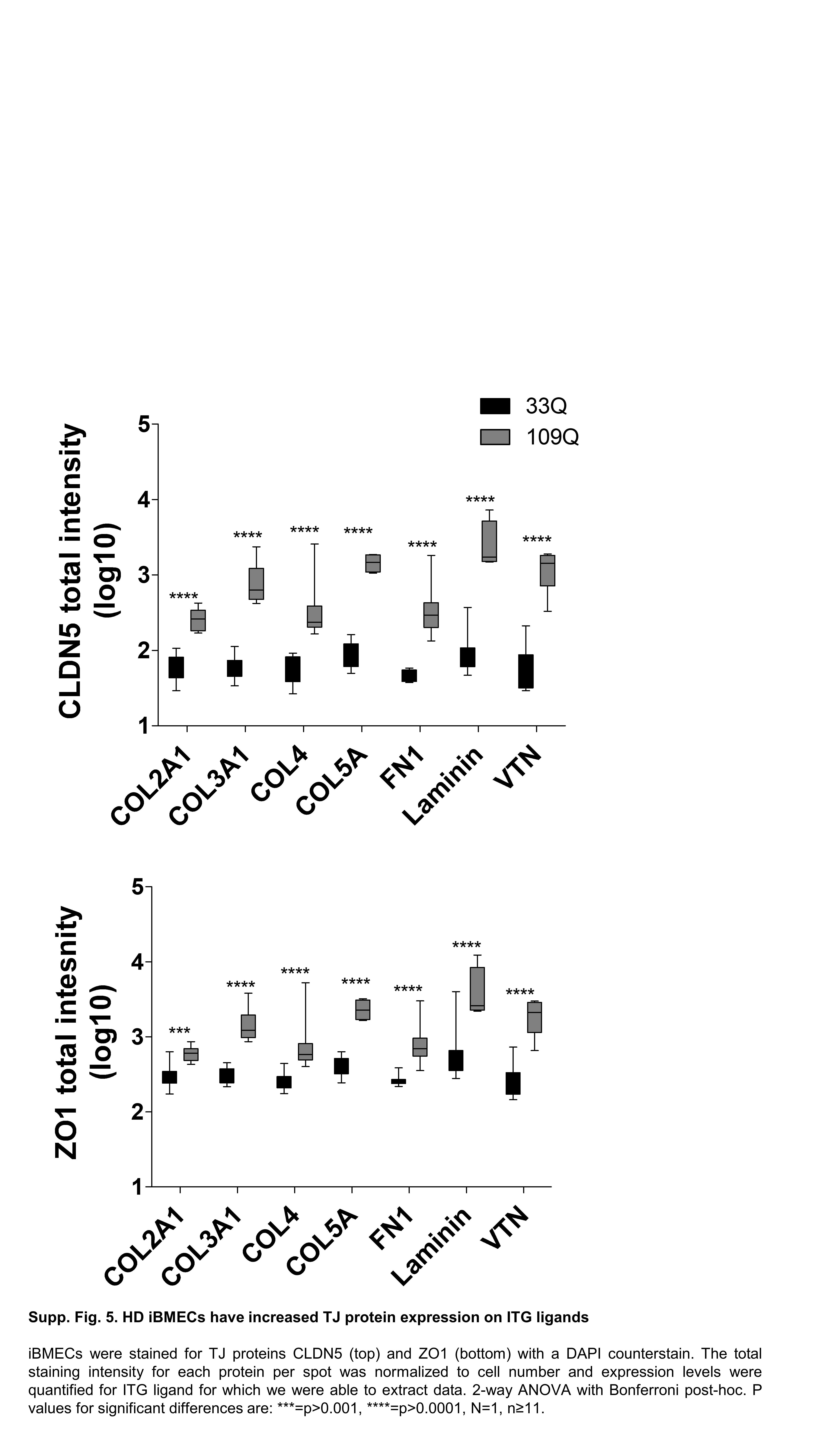

Supplement: Supp_Fig_5_ddac303 [file supp_fig_5_ddac303.zip › Supp_Fig_5_ddac303.TIF]

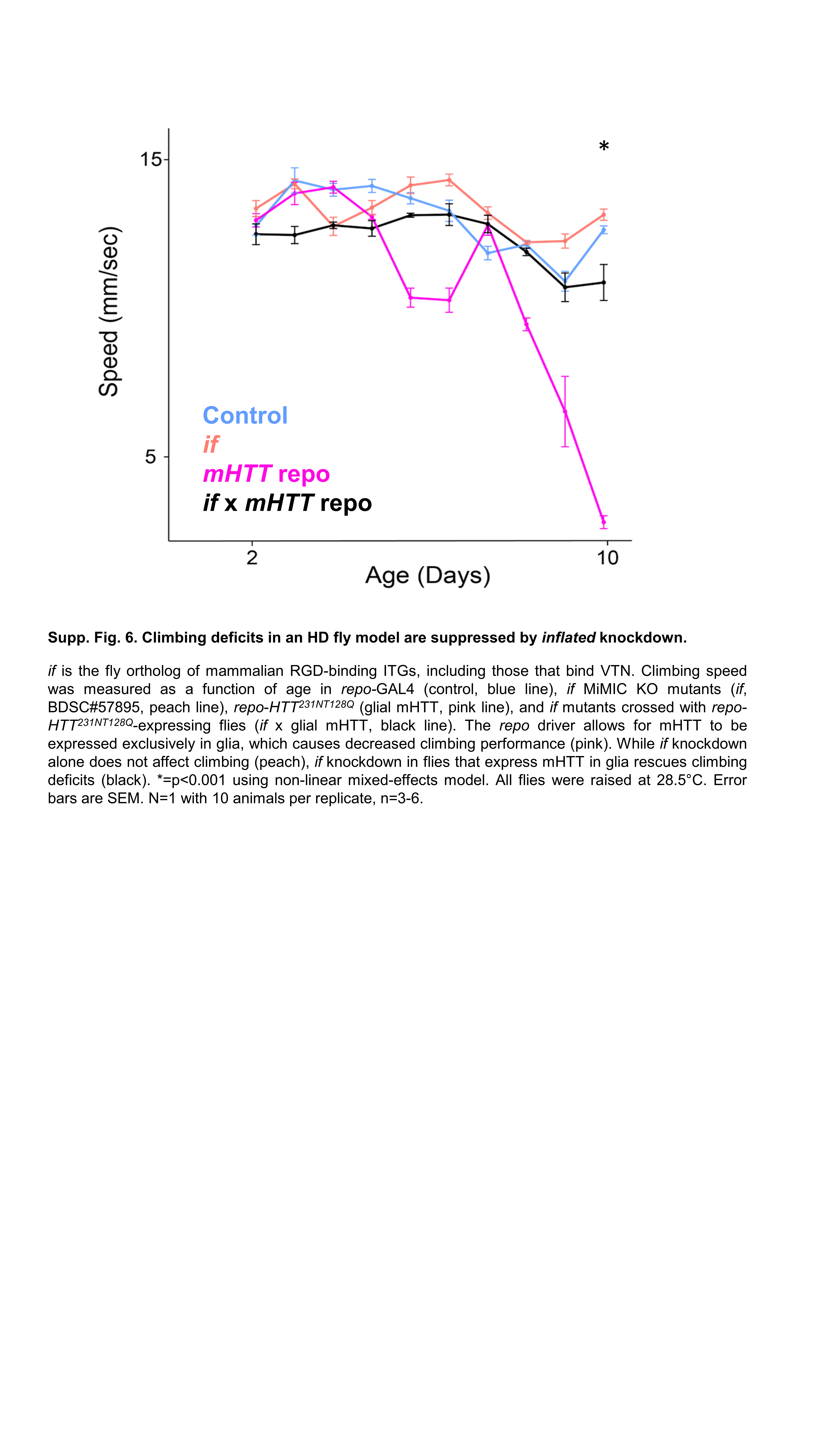

Supplement: Supp_Fig_6_ddac303 [file supp_fig_6_ddac303.zip › Supp_Fig_6_ddac303.TIF]

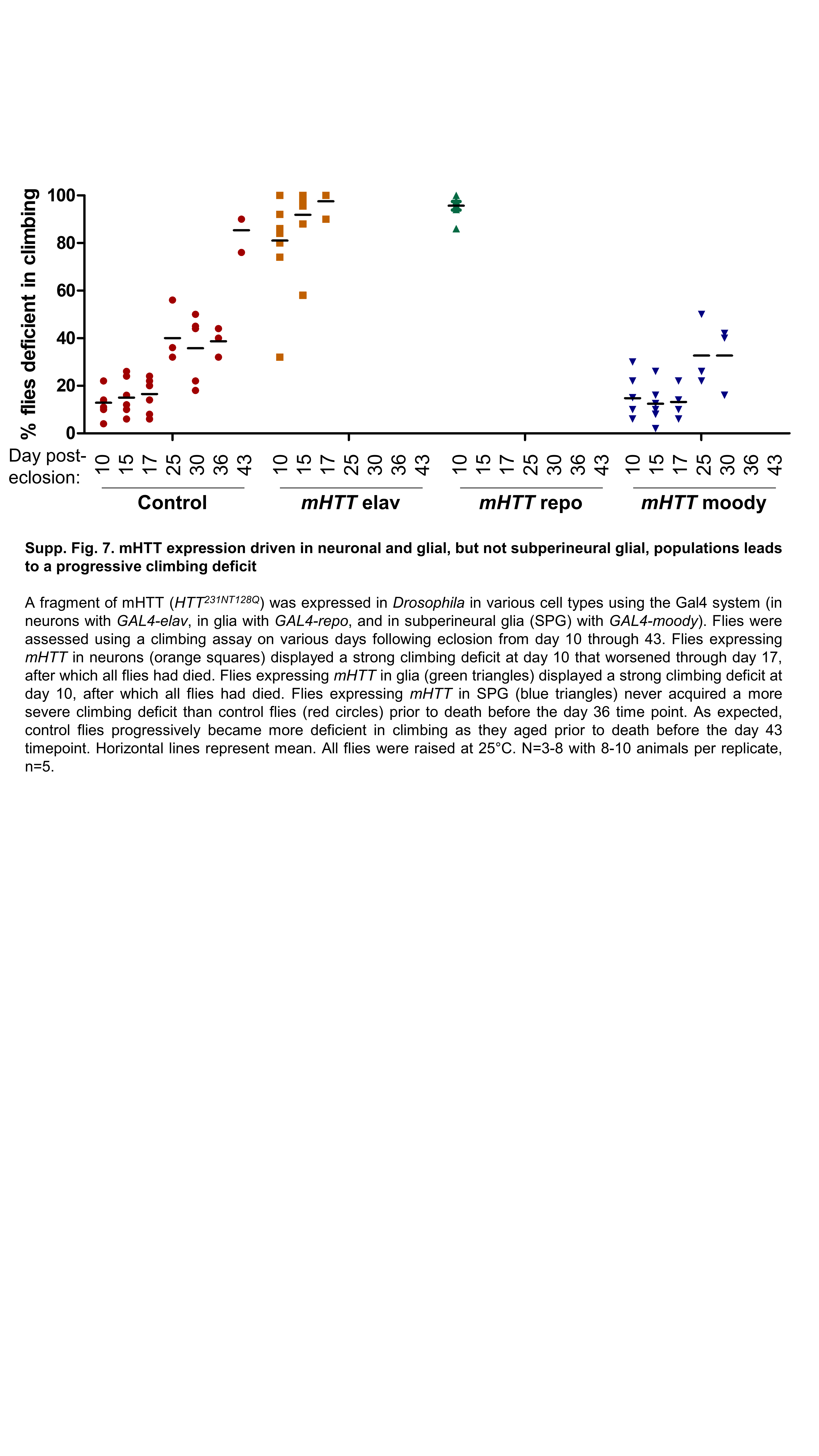

Supplement: Supp_Fig_7_ddac303 [file supp_fig_7_ddac303.zip › Supp_Fig_7_ddac303.TIF]

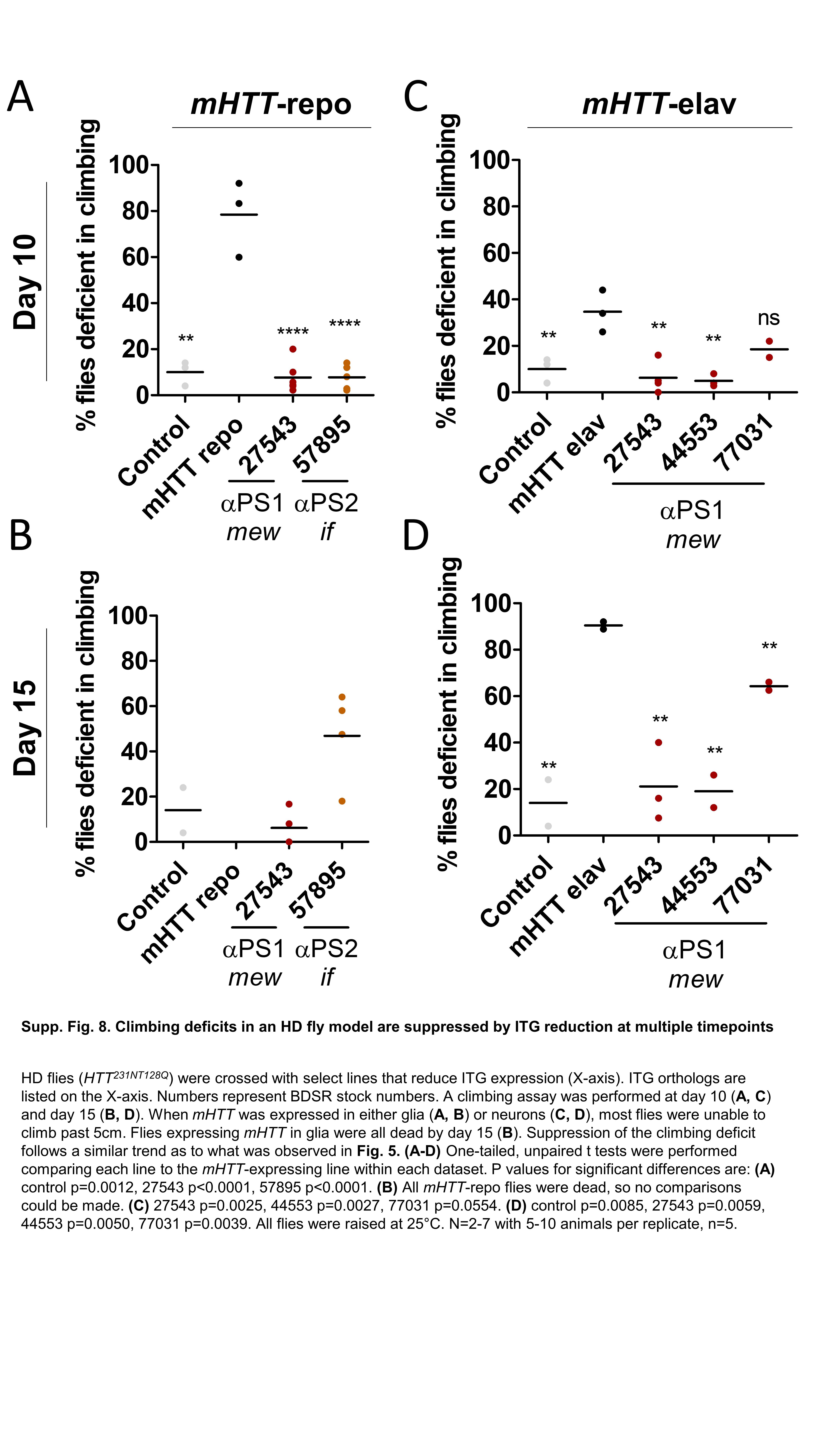

Supplement: Supp_Fig_8_ddac303 [file supp_fig_8_ddac303.zip › Supp_Fig_8_ddac303.TIF]
