# Supplementary material for: An altered extracellular matrix–integrin interface contributes to Huntington’s disease-associated CNS dysfunction in glial and vascular cells
Source: Hum Mol Genet. 2022 Dec 22;32(9):1483–96. doi: 10.1093/hmg/ddac303 (PMC10117161; doi:10.1093/hmg/ddac303)
Supplement: HMG-2022-CE-00614_R1_Supplemental_Data_(web_posting_only)_ddac303 [file hmg-2022-ce-00614_r1_supplemental_data_(web_posting_only)_ddac303.docx]

**Supplemental Figure Legends**

**Supp. Fig. 1. iPSC-derived BMEC differentiation protocol and QC images.** (**A**) Differentiation protocol for iBMECs adapted from Qian et al., Sci. Adv. 2017;3:e1701679. (**B**) iPSCs differentiated to BMECs at various timepoints throughout differentiation. Images taken at 20x on an EVOS Cell Imaging System. Scale bar = 200 μm**.** (**C**) Representative QC staining images at terminal differentiation from control (33Q, left) and HD (109Q, right) iPSCs differentiated to BMECs. All differentiations were positive for the blood-brain barrier glucose transporter GLUT1 and the tight junction markers CLDN5 and ZO1. All images were taken at 20x on a Nikon Ti-E. Scale bar = 100 μm. N=4 (33Q) and N=5 (109Q).

**Supp. Fig. 2. HD iBMECs have reduced adhesion on COL4 and VTN. (A)** A CyQUANT Cell Proliferation assay was adapted to measure cellular adhesion in 33Q and 109Q iBMECs 30 minutes following subculture. A 2-way ANOVA with a Bonferroni post-hoc test was used to demonstrate a significant reduction in adhesion in HD BMECs on collagen 4 (COL4), collagen/fibronectin (COL4/FN1), and VTN, but not FN1 alone. N=4 for 33Q and 5 for 109Q, n=4. **(B)** TEER was measured every 24 hours post-subculture as a QC metric for barrier properties. A 2-way ANOVA with a Bonferroni post-hoc test was used to demonstrate that TEER values are significantly lower in the 109Q BMECs from 48 hours on compared to the 109Q BMECs. ***<0.001, ****<0.0001. N=4 for 33Q and 5 for 109Q, n=3.

**Supp. Fig. 3. iBMEC adhesion on MEMA matrix substrates.** iBMECs were stained for TJ proteins CLDN5 (green) and ZO1 (red) with a DAPI counterstain. Images are three representative images for each cell line for each matrix. The number of technical replicates for each substrate are listed parenthetically to the right of each image. If technical replicates are listed with a slash, the number to the left is for 33Q iBMECs and the number to the right is for 109Q iBMECs. Each spot is ~300μm. Some matrices had little adhesion with either cell line **(A)** while cells adhered to others **(B)**. **(C)** 33Q and 109Q iBMECs were assessed for adhesion by counting the number of DAPI-positive cells per spot. 2-way ANOVA with Bonferroni post-hoc. P values for significant differences are: COL1 <0.000001, COL23A1 0.044490, COL2A1 <0.000001, COL3A1 <0.000001, COL4 <0.000001 COL5A1 <0.000001, FN1 <0.000001, Laminin 0.000004, VTN <0.000001. N=1, n≥11.

**Supp. Fig. 4. iBMEC adhesion on MEMA matrix substrates in combination with collagen. (A)** iBMECs stained for TJ proteins CLDN5 (green) and ZO1 (red) with a DAPI counterstain. Images are three representative images for each cell line for each matrix. The number of technical replicates for each substrate are listed parenthetically to the right of each image. Each spot is ~300μm. **(B)** 33Q and 109Q iBMECs were assessed for adhesion by counting the number of DAPI-positive cells per spot. 2-way ANOVA with Bonferroni post-hoc. P values for significant differences are: ALCAM1+COL1 <0.00001, CD44+COL1 0.000003, CDH15+COL1 <0.000001, CDH20+COL1 <0.000001, CDH8+COL1 <0.000001, CECAM6+COL1 <0.000001, DSG2+COL1 <0.000001, GAP43+COL1 <0.000001. N=1, n≥11.

**Supp. Fig. 5. HD iBMECs have increased TJ protein expression on ITG ligands.** iBMECs were stained for TJ proteins CLDN5 (top) and ZO1 (bottom) with a DAPI counterstain. The total staining intensity for each protein per spot was normalized to cell number and expression levels were quantified for ITG ligand for which we were able to extract data. 2-way ANOVA with Bonferroni post-hoc. P values for significant differences are: ***=p>0.001, ****=p>0.0001, N=1, n≥11.

**Supp. Fig. 6. Climbing deficits in an HD fly model are suppressed by *inflated* knockdown.** *if* is the fly ortholog of mammalian RGD-binding ITGs, including those that bind VTN. Climbing speed was measured as a function of age in *repo-*GAL4 (control, blue line), *if* MiMIC KO mutants (*if*, BDSC#57895, peach line), *repo*-*HTT231NT128Q* (glial mHTT, pink line), and *if* mutants crossed with *repo- HTT231NT128Q*-expressing flies (*if* x glial mHTT, black line). The *repo* driver allows for mHTT to be expressed exclusively in glia, which causes decreased climbing performance (pink). While *if* knockdown alone does not affect climbing (peach), *if* knockdown in flies that express mHTT in glia rescues climbing deficits (black). *=p<0.001 using non-linear mixed-effects model. All flies were raised at 28.5°C. Error bars are SEM. N=1 with 10 animals per replicate, n=3-6.

**Supp. Fig. 7. mHTT expression driven in neuronal and glial, but not subperineural glial, populations leads to a progressive climbing deficit.** A fragment of mHTT (*HTT231NT128Q*) was expressed in *Drosophila* in various cell types using the Gal4 system (in neurons with *GAL4-elav*, in glia with *GAL4-repo*, and in subperineural glia (SPG) with *GAL4-moody*). Flies were assessed using a climbing assay on various days following eclosion from day 10 through 43. Flies expressing *mHTT* in neurons (orange squares) displayed a strong climbing deficit at day 10 that worsened through day 17, after which all flies had died. Flies expressing *mHTT* in glia (green triangles) displayed a strong climbing deficit at day 10, after which all flies had died. Flies expressing *mHTT* in SPG (blue triangles) never acquired a more severe climbing deficit than control flies (red circles) prior to death before the day 36 time point. As expected, control flies progressively became more deficient in climbing as they aged prior to death before the day 43 timepoint. Horizontal lines represent mean. All flies were raised at 25°C. N=3-8 with 8-10 animals per replicate, n=5.

**Fig. 5. HD-associated CNS deficits are suppressed when ITG expression is reduced in glial, but not neuronal, cell populations.** An HD fragment *Drosophila* model (*HTT231NT128Q*) was crossed with lines that reduce expression of various ITGs (X-axis) expressed using the Gal4 system. ITG orthologs are listed on the X-axis. Numbers represent BDSC stock numbers. A climbing assay was performed **(A)** at day 10 when *mHTT* was expressed in glia and **(B)** day 15 when *mHTT* was expressed in neurons. When *mHTT* was expressed in either glia (grey dots, **A**) or neurons (grey dots, **B**), most flies were unable to climb past 5cm. When ITG mutants were crossed with flies that express *mHTT* only in glia (black dots, **A**), the climbing deficit was largely suppressed. When ITG mutants were crossed with flies that express *mHTT* only in neurons (black dots, **B**), the climbing deficit is less suppressed overall. Horizontal lines represent mean. One tailed, unpaired t tests were performed comparing each line to the *mHTT*-expressing line within each dataset. P values for significant differences are: **** = p<0.0001, *** = p=0.001. **(A)** 57895 p=0.0174, 68158 p=0.3103. **(B)** 27543 = p=0.0002, 44553 p=0.0409, 27544 p=0.0195, 28535 p=0.0044, 27735 p=0.0006. All flies were raised at 25°C. N=3-9 with 8-10 animals per replicate, n=5.

**Supp. Fig. 8. Climbing deficits in an HD fly model are suppressed by ITG reduction at multiple timepoints.** HD flies (*HTT231NT128Q*) were crossed with select lines that reduce ITG expression (X-axis). ITG orthologs are listed on the X-axis. Numbers represent BDSR stock numbers. A climbing assay was performed at day 10. (**A, C**) and day 15 (**B, D**). When *mHTT* was expressed in either glia (**A, B**) or neurons (**C, D**), most flies were unable to climb past 5cm. Flies expressing *mHTT* in glia were all dead by day 15 (**B**). Suppression of the climbing deficit follows a similar trend as to what was observed in **Fig. 5**. **(A-D)** One-tailed, unpaired t tests were performed comparing each line to the *mHTT*-expressing line within each dataset. P values for significant differences are: **(A)** control p=0.0012, 27543 p<0.0001, 57895 p<0.0001. **(B)** All *mHTT*-repo flies were dead, so no comparisons could be made. **(C)** 27543 p=0.0025, 44553 p=0.0027, 77031 p=0.0554. **(D)** control p=0.0085, 27543 p=0.0059, 44553 p=0.0050, 77031 p=0.0039. All flies were raised at 25°C. N=2-7 with 5-10 animals per replicate, n=5.
